# In *Campylobacter jejuni*, a new type of chaperone receives heme from ferrochelatase

**DOI:** 10.3389/fgene.2023.1199357

**Published:** 2023-06-21

**Authors:** Jordi Zamarreño Beas, Marco A. M. Videira, Val Karavaeva, Frederico M. Lourenço, Mafalda R. Almeida, Filipa Sousa, Lígia M. Saraiva

**Affiliations:** ^1^ Instituto de Tecnologia Química e Biológica António Xavier, Universidade Nova de Lisboa, Oeiras, Portugal; ^2^ Department of Functional and Evolutionary Ecology, University of Vienna, Wien, Austria

**Keywords:** heme, *Campylobacter*, biogenesis, chaperone, ferrochelatase

## Abstract

Intracellular heme formation and trafficking are fundamental processes in living organisms. Bacteria and archaea utilize three biogenesis pathways to produce iron protoporphyrin IX (heme *b*) that diverge after the formation of the common intermediate uroporphyrinogen III (uro’gen III). In this study, we identify and provide a detailed characterization of the enzymes involved in the transformation of uro’gen III into heme in *Campylobacter jejuni*, demonstrating that this bacterium utilizes the protoporphyrin-dependent (PPD) pathway. In general, limited knowledge exists regarding the mechanisms by which heme *b* reaches its target proteins after this final step. Specifically, the chaperones necessary for trafficking heme to prevent the cytotoxic effects associated with free heme remain largely unidentified. In *C. jejuni*, we identified a protein named CgdH2 that binds heme with a dissociation constant of 4.9 ± 1.0 µM, and this binding is impaired upon mutation of residues histidine 45 and 133. We demonstrate that *C. jejuni* CgdH2 establishes protein–protein interactions with ferrochelatase, suggesting its role in facilitating heme transfer from ferrochelatase to CgdH2. Furthermore, phylogenetic analysis reveals that *C. jejuni* CgdH2 is evolutionarily distinct from the currently known chaperones. Therefore, CgdH2 is the first protein identified as an acceptor of intracellularly formed heme, expanding our knowledge of the mechanisms underlying heme trafficking within bacterial cells.

## 1 Introduction

*Campylobacter* (*C*.) *jejuni* is a Gram-negative microaerophilic Epsilon proteobacterium responsible for a high percentage of diarrheal infections worldwide, often resulting from the consumption of poultry meat contaminated with antibiotic-resistant strains (Jadeja and Worrich, 2022).

Heme is an iron-porphyrin cofactor that is essential to several life-associated cellular processes, including respiration, signaling, RNA processing, redox catalysis, stress responses, and cellular differentiation. Similar to most other living systems, bacteria require heme for normal functioning and usually express systems dedicated to synthesizing and/or acquiring heme from the host.

Most prokaryotes have the ability to endogenously synthesize heme *b* (aka heme) through a multi-step process named the heme biosynthesis pathway (Dailey et al., 2017). Three pathways are so far known to occur in bacteria and archaea: the protoporphyrin-dependent (PPD) pathway, the coproporphyrin-dependent (CPD) pathway, and the siroheme-dependent (SHD) pathway. All pathways share as the first common precursor the 5-aminolevulinic acid (ALA) molecule, which is converted to uroporphyrinogen III (uro’gen III) through the action of three enzymes: PbgS, HmbS, and UroS (Heinemann et al., 2008). The conversion of uro’gen III to heme occurs according to one of three pathways depending on the organism, which are described in detail in several reviews (e.g., Dailey and Medlock, 2022; Zamarreño Beas et al., 2022).

Most Gram-negative bacteria utilize the PPD pathway, formerly known as the classic pathway, which was the first identified heme biosynthesis route. The PPD pathway begins with the decarboxylation of the acetic acid groups of uro’gen III forming coproporphyrinogen III, followed by the decarboxylation of two of the propionic acid chains of coproporphyrinogen III to produce protoporphyrinogen IX. The first decarboxylation reaction is catalyzed by the enzyme uro’gen III decarboxylase (UroD). The second reaction is carried out by coproporphyrinogen decarboxylase (CgdC) that requires molecular oxygen, or by the oxygen-independent coproporphyrinogen dehydrogenase CgdH (previously named HemN or HemZ). In addition to the *bona fide* CgdHs that possess coproporphyrinogen III dehydrogenase activity (Layer et al., 2003), bacteria may contain other *cgdH*-like genes with different functions (Cheng et al., 2022). This is the case of the HemW-like proteins, which are chaperons involved in heme transfer, or possess enzyme activities such as methyltransferases and cyclopropanases (Haskamp et al., 2018; Cheng et al., 2022). Protoporphyrinogen IX, formed by CgdH, is then converted to protoporphyrin IX by one of the membrane-associated enzymes PgoX, PgdH1, and PgdH2. PgoX is an oxygen-dependent proto’gen oxidase that contains tightly non-covalently bound FAD, which was studied in *Acinetobacter* and *Synechocystis sp*. (Kobayashi et al., 2014; Dailey et al., 2017). PgdH1 and PgdH2, which share low amino sequence similarity, are considered protoporphyrinogen dehydrogenases as they require anoxic conditions to produce protoporphyrin IX. PgdH1 is an FMN-containing protein that interacts with the cellular respiratory chain (Möbius et al., 2010). Although PgdH2 is the least-characterized enzyme and it is not known whether it requires specific cofactors, it is the most common protoporphyrinogen dehydrogenase in Gram-negative heme-synthesizing bacteria (with an occurrence of >60%). Finally, in the last step of the PPD pathway, iron is inserted into protoporphyrin IX to produce protoheme IX (heme *b*) via the enzyme protoporphyrin ferrochelatase (PpfC) (Kobayashi et al., 2014).

*C. jejuni* also contains a heme uptake system, which includes the TonB-dependent heme receptor (ChuA), the heme ABC transporter permease (ChuB), the heme ABC transporter ATP-binding protein (ChuC), the periplasmic heme-binding protein (ChuD), and the heme oxygenase (ChuZ). The gene encoding ChuZ is transcribed divergently from the *chuABCD* operon. This heme import system contributes to the virulence of *C. jejuni* (Ridley et al., 2006; Johnson et al., 2016; Richard et al., 2019). Although the heme uptake in *C. jejuni* has been extensively investigated, the specific type of heme biosynthesis pathway operating in this bacterium has to be determined yet.

In this study, we have identified how *C. jejuni* produces heme intracellularly through the detailed characterization of the enzymes forming the pathway that converts uro’gen III to heme. Despite the crucial role of intracellular heme trafficking and the need for tight regulation to prevent heme toxicity, the mechanisms underlying heme transport and delivery are not well understood. In our study, we also found a novel heme-binding protein that can receive heme from ferrochelatase, the final enzyme in the PPD pathway.

## 2 Materials and methods

### 2.1 Growth of strains and gene cloning

The strains used in this work are listed in Supplementary Table S1. *Escherichia* (*E*.) *coli* strains were grown under aerobic conditions in Luria–Bertani broth (Roth) at 37°C and 150 rpm. The *C. jejuni* NCTC 11168 strain was first plated in Muller–Hinton agar, grown in Muller–Hinton broth in flasks filled with 1/10 of the volume, and incubated in a microaerobic environment (83.3% N_2_, 7.1% CO_2_, 3.6% H_2_, and 6% O_2_). Cultures were supplied with appropriate antibiotics, such as ampicillin (50 μg mL^−1^), kanamycin (50 μg mL^−1^), and chloramphenicol (30 μg mL^−1^).

*C. jejuni* NCTC 11168 genes encoding UroD (*cj124*3), CgdH1 (*cj0992c*), CgdH2 (*cj0363c*), CgdH3(*cj0580c*), PgdH2 (*cj0362*), and PpfC (*cj0503c*) were amplified by PCR from genomic DNA, extracted according to the manufacturer’s instructions (QIAprep Spin Miniprep Kit), using the Phusion High-Fidelity DNA Polymerase (Thermo Fisher) and the primers described in Supplementary Table S2. After restriction enzyme cleavage, DNA fragments were cloned into the chosen plasmids with the use of T4 DNA ligase.

We used the pPR-IBA2 vector (IBA) to produce the N-terminal Strep-tag fused proteins and pET-23b for C-terminal His-tag fused proteins. The correct tag insertions were confirmed by DNA sequencing. Plasmids pPR-IBA2 containing *C. jejuni uroD* or *C. jejuni ppfC* were used in complementation assays (Supplementary Table S3. *E. coli ΔppfC* and *E. coli ΔuroD* mutant strains were obtained from the National BioResource Project (NBRP) at the National Institute of Genetics (NIG; https://shigen.nig.ac.jp/ecoli/strain/). These strains were made competent and transformed with plasmid DNA by heat shock treatment. Cells were grown overnight in LB supplemented with hemin (10 µM). After ∼16 h of growth, 1 mL of the overnight culture was centrifuged at 1,700 × *g*, resuspended three times in PBS, and then diluted 1:10 six times. From each dilution, 5 mL were spotted in LB agar supplemented, or not, with hemin at the indicated concentrations. The strains and plasmids used in this study are presented in Supplementary Table S3.

### 2.2 Production and purification of recombinant proteins

Cells of *E. coli* BL21STAR(DE3)pLysS (Novagen) transformed with pPR-IBA2-*uroD-strep* plasmid, pET23b-*cgdH*1, and pET23b-*cgdH2-his* were grown overnight, diluted 1:100 fold in fresh LB containing ampicillin. The ratio 2/5 of liquid medium/flask volume was used. Cells were grown at 37°C 150 rpm to OD_600nm_∼0.6. At this point, isopropyl β-D-1-thiogalactopyranoside (IPTG) was added to induce expression of UroD (50 µM IPTG), CgdH1, and CgdH2 (500 µM IPTG). The temperature was lowered to 20°C, and cells were grown for 16–20 h. Cells were harvested by centrifugation (11,000 × *g*, 10 min, 4°C), and pellets were resuspended in 100 mM Tris-HCl, pH 8 (buffer A) with 150 mM NaCl. Cells were disrupted in a French press operating at 1,000 Psi and centrifuged (42,000 × *g*, 40 min, 4°C).

For the purification of *C. jejuni* UroD-Strep, the supernatant, which had been previously passed through a 0.45-µm filter, was applied onto Strep-Tactin Superflow^®^ high-capacity resin (Iba^®^), that had been equilibrated with buffer A. After washing the resin with at least three column volumes of buffer A, the proteins were eluted with buffer A containing 150 mM NaCl and 2.5 mM desthiobiotin. After elution, all subsequent steps were performed under anaerobic conditions. Buffer exchange to 50 mM Tris-HCl, pH 8 (buffer B) was achieved using a PD-10 column, and the protein was concentrated using a diaflow with suitable cut-off membranes (EMD Millipore Corporation, Billerica, Massachusetts).

For the purification of *C. jejuni* CgdH1-His and CgdH2-His, the supernatants were separately applied to Chelating Sepharose™ fast flow resin columns (GE Healthcare, Carnaxide, Portugal) charged with Ni^2+^ and previously equilibrated with four column volumes of buffer B containing 500 mM NaCl and 10 mM imidazole. After washing the columns with buffer B containing 500 mM NaCl, buffers with increasing concentrations of imidazole were applied (10–500 mM), and His-tag CgdH1 and CgdH2 proteins were eluted at 400 mM imidazole.

For the production of *C. jejuni* PpfC-Strep enzyme, 1% of a pre-culture of *E. coli* BL21STAR(DE3)pLysS (Novagen) cells harboring the pPRIBA2-ppfC-strep was used to inoculate fresh LB media containing ampicillin. When the cells reached an OD600 of ∼0.7, 500 μM of IPTG was added, and the cells were further grown for 3.5 h at 30°C at 150 rpm. Cells were harvested by centrifugation (11,000 × *g*, 10 min, 4°C), and the pellets were resuspended in buffer B supplemented with 150 mM NaCl, 10% glycerol, and 1% Triton X-100. Cells were disrupted in a French press operating at 1,000 Psi, and centrifuged at 48,000 × *g*, at 4°C, for 30 min. The lysates were added to a Strep-Tactin Sepharose column and washed with buffer B supplemented with 400 mM NaCl, 10% glycerol, and increasing concentrations of desthiobiotin. The protein was eluted with buffer B containing 5 mM desthiobiotin. All protein fractions were dialyzed in buffer B with 100 mM NaCl, and their purity was confirmed by SDS-PAGE.

For the iron–sulfur (Fe–S) center reconstitution assays of the purified *C. jejuni* His-tagged CgdH1 and CgdH2, the proteins were diluted to 200 µM in buffer B containing 5 mM DTT, 150 mM NaCl, 20% glycerol, and an eight-fold molar excess of FeCl_3_. After incubation for 5 min, lithium sulfide was added at the same molar concentration as iron, and the mixture was incubated at 4°C for 3 h. Unbound iron and sulfide were removed using a PD-10 desalting column (GE Healthcare). The formation of the Fe–S center was confirmed by UV-visible spectroscopy (Supplementary Figure S2).

### 2.3 Biochemical assays

Porphobilinogen, coproporphyrin III, protoporphyrin IX, and hemin were purchased from Frontier Scientific. Freshly prepared enzymes were used all the time, and the assays were performed in a Coy model A-2463 Belle Technology anaerobic chamber, which housed a Shimadzu UV-1800 spectrophotometer. Porphyrins were detected by their spectral absorbance bands localized at 390 and 410 nm. Unless otherwise indicated, reactions were carried out in buffer B at room temperature.

*C. jejuni* UroD activity was determined by a linked protein assay that allowed the production of uroporphyrinogen III (uro’gen III), as described previously (Lobo et al., 2015). Briefly, reaction mixtures carried out in buffer B (final volume of 2 mL) containing 2 mg of *Desulfovibrio* (*D*.) *vulgaris* PbgS, 2 mg of *D. vulgaris* UroS, and 1.5 mg of porphobilinogen were prepared in the absence (control reaction) and in the presence of 1.5 mg of *C. jejuni* UroD, incubated overnight, and oxidized by the addition of 0.1 M HCl. Following a centrifugation step (10,000 × *g*, 10 min) to remove the protein precipitates, the formation of the substrate uro’gen III (present in the control reaction) and the product copro’gen III (resulting from the activity of UroD) were detected in their oxidized form, namely, uroporphyrin III and coproporphyrin III, using UV-visible spectroscopy and HPLC-MS analysis. For the latter, samples were resolved on an Ace5-AQ column attached to an Agilent 1100 series HPLC equipped with a diode array detector and coupled to a micrOTOF-Q II (Bruker) mass spectrometer. Separation of the products was achieved by applying a binary gradient of 0.1% TFA (solvent A) and acetonitrile (solvent B), at a flow rate of 0.2 mL min^−1^. The column was initially equilibrated with 80% of solvent A and 20% of solvent B. The samples were injected, and a concentration gradient of solvent B reached up to 100% within 50 min.

For *C. jejuni* CgdH1 and CgdH2 activity assays, copro’gen III was first prepared in a reaction mixture containing *D. vulgaris* PbgS, *D. vulgaris* UroS, porphobilinogen, and *C. jejuni* UroD in concentrations similar to those described previously. The concentration of the copro’gen III formed was determined by adding 0.1 M HCl and measuring the absorbance at 548 nm (ε_548_ = 16.8 mM^−1^ cm^−1^). In separate reactions, each *C*. *jejuni* CgdH protein variant with Fe–S reconstituted (∼20 μM) was incubated with 10 μM of copro’gen III, 1 mM of S-adenosyl-L-methionine (SAM), 0.5 mM of NADH, and 30 μL of *E. coli* BL21STAR(DE3)pLysS cell extract (10 mg/mL of protein concentration). The reaction mixture was incubated overnight at room temperature in the anaerobic glove box. The resulting samples were treated with 0.6 M HCl and centrifuged at 10,000 × *g* for 10 min to remove protein precipitates. The porphyrins formed in the reaction were separated on an Ace5-AQ C18 column attached to a Hitachi LaChrom Elite HPLC equipped with a diode array detector (VWR, Alfragide, Portugal) using the same solvents and gradients above previously.

Proto’gen IX was prepared by the reduction of protoporphyrin IX using a sodium mercury amalgam. Approximately 3 mg of protoporphyrin IX was solubilized with three drops of 30% ammonium hydroxide and dissolved in 5 mL of freshly prepared 10 mM KOH. The concentration was determined, and the porphyrin was diluted to 50 μM in a 2 mL final volume. Sodium mercury amalgam (5%) from Sigma was added to the solution, which was then sealed and degassed with nitrogen, and 100 μL of 1 M Tris-HCl, pH 8 was added. The reaction was kept in the anaerobic chamber protected from light. The solution containing proto’gen IX was filtered, and the concentration of the compound was determined spectroscopically in 0.1 M HCl (ɛ_408_ = 297 mM^−1^ cm^−1^).

For the determination of proto’gen IX oxidase activity, reaction mixtures were prepared in buffer B with a final volume of 200 μL. The mixtures contained 500 nM proto’gen IX, 150 mM NaCl, 20% glycerol, 5 mM DTT, and 0.3 mg of the membrane fraction of *E. coli* BL21STAR(DE3)pLysS cell extracts either containing *C. jejuni* PgdH2 or lacking this protein (empty pPR-IBA2; negative control). Samples were prepared in black 96-well plates (Greiner), and the fluorescence was monitored for 20 min. The formation of protoporphyrin IX was evaluated using a 390 nm excitation filter and a 630 nm emission filter in a Fluorostar Optima plate reader (BMG Laboratories) under aerobic conditions. The fluorescence intensity units were converted to concentration by interpolation of a calibration curve obtained with protoporphyrin IX standards. The auto-oxidation of the substrate was subtracted from the oxidation generated by the membrane fractions. The initial rate was calculated from the linear region of the graph.

For the ferrochelatase activity, protoporphyrin IX was prepared as described previously (Lobo et al., 2015). Briefly, ∼1 mg of protoporphyrin IX was solubilized in 1–3 drops of 25% NH_4_OH and subsequently diluted in 500 μL Triton X-100 and 4.5 mL water. The concentration of protoporphyrin IX was determined in 2.6 M HCl by measuring the absorbance at 408 nm (ε = 297 mM^−1^ cm^−1^). The reaction mixtures contained 10 μM of protoporphyrin IX and 30 μg of *C. jejuni* PpfC in buffer B supplemented with 20 μM of (NH_4_)_2_Fe(SO_4_)_2_, 5 mM DTT, 150 mM NaCl, and 20% glycerol. *C. jejuni* PpfC activity was assayed by following the decrease in the amount of the substrate protoporphyrin IX in solution, which was monitored at 408 nm.

### 2.4 Heme binding, pull-down, and heme transfer assays

For the heme-binding assay, the soluble cell fraction containing CgdH2 (prepared as described previously) was incubated with 300 μM of hemin (Roth), for 1 h at room temperature. The protein solution was loaded onto a Chelating Sepharose™ fast flow column (GE Healthcare) equilibrated with 20 mM Tris-HCl, pH 8, containing 500 mM NaCl, and 10 mM imidazole. The resin was washed with increasing concentrations of imidazole, and the protein was eluted with 0.5 M imidazole. To remove any unbound hemin, the pure protein was loaded onto a PD-10 column (GE Healthcare) and washed with 20 mM Tris-HCl, pH 8 buffer. The heme bound to the protein was quantified by measuring the absorption spectra of pyridine hemochromes (Berry and Trumpower, 1987).

The heme titration experiment was performed essentially as described previously (Lobo et al., 2017). Briefly, purified *C. jejuni* apo-CgdH2 was diluted to the final concentration of 5 μM in buffer B and loaded into a magnetically stirred quartz (10 mm) cuvette. Increasing concentrations of hemin (from 1 to 20 μM), diluted in 0.1 M NaOH, were added to the protein solution. The procedure was also performed using a similar solution mixture that did not contain protein, serving as a control reaction. The titration was monitored by UV-visible spectroscopy in a Shimadzu UV-1700 spectrophotometer at room temperature. The binding stoichiometry and affinities were determined by plotting the absorbance difference spectra measured at 416 nm.

For the pull-down assay, apo-PpfC-Strep was incubated for 30 min with apo-CgdH2-His in buffer B at 4°C. The apo-PpfC-Strep, apo-CgdH2-His, and a mixture of apo-PpfC-Strep with apo-CgdH2-His were loaded separately onto Strep-Tactin Sepharose resin-containing columns. The resins were washed with three volumes of buffer B containing 400 mM NaCl and 10% glycerol and eluted with two volumes of the same buffer supplemented with 5 mM desthiobiotin.

For the heme transfer assays, a cell lysate expressing *C. jejuni* PpfC-Strep was prepared as described previously. Prior to the purification step, the cell lysate was incubated with ∼100 μM of hemin for 1 h at room temperature, after which it was loaded onto the Strep-Tactin Sepharose column, and purification followed the protocol indicated previously. The purified PpfC was isolated, bound to heme, and designated as He-PpfC-Strep. To test the heme transfer between the two *C. jejuni* proteins, purified He-PpfC-Strep was mixed with purified apo-CgdH2-His for 1 h at room temperature in buffer B. The apo-CgdH2-His (20 μM), He-PpfC-Strep (10 μM), and the mixture of the two were separately loaded onto Strep-Tactin^®^ Sepharose resins. The resins were then washed with buffer B containing 400 mM NaCl and 10% glycerol, with or without supplementation of 5 mM desthiobiotin. Fractions were analyzed by SDS-PAGE (stained with Coomassie Blue) and UV-visible spectroscopy.

### 2.5 Bioinformatic and phylogeny analysis

The Basic Local Alignment search tool (BLAST) was used to search genomes for proteins involved in tetrapyrrole biosynthesis, and sequence alignments were performed with ClustalW using the predefined parameters.

The three-dimensional protein structures were predicted using the software AlphaFold2 through MMseqs2 and its predefined settings (Mirdita et al., 2022). The similarity between the structures was determined using PyMOL software (Schrödinger, 2020). Data quality was evaluated through the root mean square deviation (RMSD) value that reflects the average distance between the atoms of each protein structure, with values decreasing with the increasing fitting between the two structures. Structures were visualized using PyMOL and utilized for *in silico* protein docking studies.

The subgroup of the Radical SAM superfamily that includes the anaerobic coproporphyrinogen-III oxidase-like enzymes from the Structure Function Linkage Database network (repnet.sg1065.th50.pE20.mek250.xgmml) (Akiva et al., 2014) was used in Cytoscape (v3.8.0; following the protocol described in www.sfld.rbvi.ucsf.edu) to distinguish the three CgdH proteins. This network distribution is based on the enzyme sequence, structure, and molecular function. The database includes cyclopropanases, heme degradation proteins (HutW/ChuW-like), HemN-like clusters with heat shock encoded genes, HemN-like clusters with nucleoside-triphosphate RdgB, and oxygen-independent coproporphyrinogen-III oxidases 1 and 2 (HemN and HemZ-like). The nodes were organized based on the “rep-net mean” value, and nodes with a −log10(E-value) below 60 are not considered in the analysis.

A cluster analysis was performed for the three CgdH proteins using the Structure Function Linkage Database (SFLD) of the Radical SAM (RSM) proteins superfamily, subgroup of the coproporphyrinogen III oxidase-like proteins (1,045 nodes, ∼65,000 protein GIs), and each node represents a set of proteins that share at least 50% identity, i.e., it is a group of similar sequences (a total of ∼100 nodes). Edges between nodes indicate a mean BLAST E-value among all pair sequences in these nodes with an e-value lower than 1e-60 (Akiva et al., 2014).

For phylogeny analysis, protein sequences selected by Cheng et al. (2022) were retrieved from the NCBI database based on accession numbers and mapped to NCBI Taxonomy. CgdH1, CgdH2, and CgdH3 sequences were also included in the analysis (Supplementary Table S4). A multiple sequence alignment was performed using Clustal Omega [https://www.ebi.ac.uk/Tools/msa/clustalo/ (Sievers et al., 2011), with Max guide tree operations = 5 and Max HMM iterations = 5, and then trimmed using trimAl v1.3 (Capella-Gutiérrez et al., 2009)] through the Phylemon 2 webserver using the following parameters: minimum percentage of positions to conserve = 60 and gap threshold = 0.05. A maximum likelihood phylogenetic reconstruction was carried out in the IQ-TREE webserver (Trifinopoulos et al., 2016) considering 1,000 ultrafast bootstraps and best model selection. The phylogenetic reconstruction was rooted by using the minimal ancestor deviation (MAD) method [version 2.22, (Tria et al., 2017)] with a modified script to keep bootstrap values. FigTree was used for annotations and analysis (v1.4.4, http://tree.bio.ed.ac.uk/software/figtree/).

## 3 Results

**FIGURE 1:**
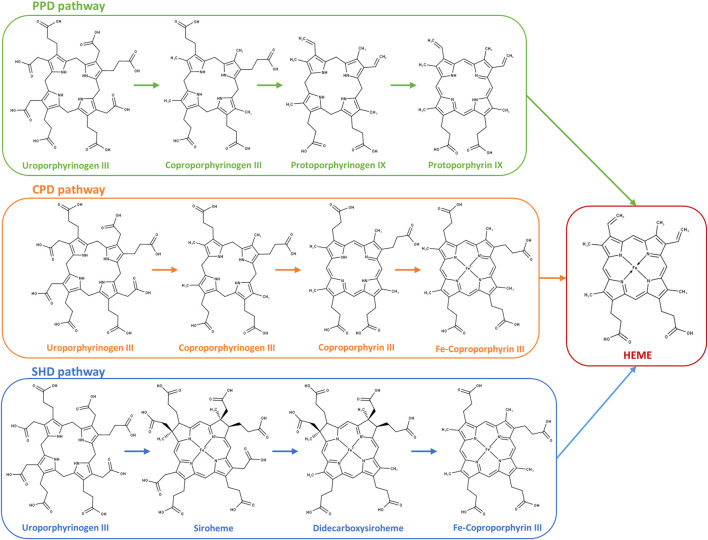
Heme biosynthesis pathways in prokaryotes. Protoporphyrin-dependent (PPD) pathway; coproporphyrin-dependent (CPD) pathway; and siroheme-dependent (SHD) pathway depicted from uroporphyrinogen III to the final product heme. PPD pathway: 1) decarboxylation of the four acetate side chains of uroporphyrinogen III to methyl groups forming coproporphyrinogen III; 2) oxidative decarboxylation of the propionate substituents on the pyrrole rings A and B of coproporphyrinogen III generating the corresponding vinyl groups in protoporphyrinogen IX; 3) oxidation of protoporphyrinogen IX to protoporphyrin IX; and 4) insertion of ferrous iron into protoporphyrin IX for heme formation. CPD pathway: 1) decarboxylation of uroporphyrinogen III to coproporphyrinogen III (identical to the first reaction of the PPD pathway); 2) oxidation of coproporphyrinogen III to coproporphyrin III; 3) insertion of ferrous iron into the coproporphyrin III macrocycle forming Fe–coproporphyrin III; and 4) oxidative decarboxylation of the propionate side chains in pyrrole rings A and B of Fe–coproporphyrin III to yield the corresponding vinyl groups of heme. SHD pathway: 1) uroporphyrinogen III is methylated to precorrin-2, oxidation of precorrin-2 generates sirohydrochlorin, and by insertion of iron, siroheme is formed; 2) siroheme undergoes decarboxylation of the acetic side chains to give didecarboxysiroheme; 3) the loss of two acetic side chains generates Fe–coproporphyrin III (coproheme); and 4) in the final step occurs the loss of the carboxylic acid groups of the propionate side chains to generate heme.

The heme biosynthesis pathways known to date utilize uroporphyrinogen III (uro’gen III) as a common precursor that is converted to heme *b* through three possible pathways that vary according to the organism (Figure 1). In this work, we sought to identify which pathway *C. jejuni* uses to synthesize heme from uro’gen III. We searched the *C. jejuni* NCTC 11168 genome to identify genes encoding proteins putatively involved in the conversion of uro’gen III to heme *b* and found proteins that share significant amino acid sequence similarity with bacterial heme-biosynthesis-related proteins. *C. jejuni* genes were cloned, the recombinant proteins were produced and biochemically characterized, and their structures were modelled using AlphaFold2 (Mirdita et al., 2022). The porphyrin products of the enzymatic assays were detected by UV-visible spectroscopy and high-performance liquid chromatography-mass spectrometry (HPLC-MS).

### 3.1 Uro’gen III to copro’gen III

**FIGURE 2:**
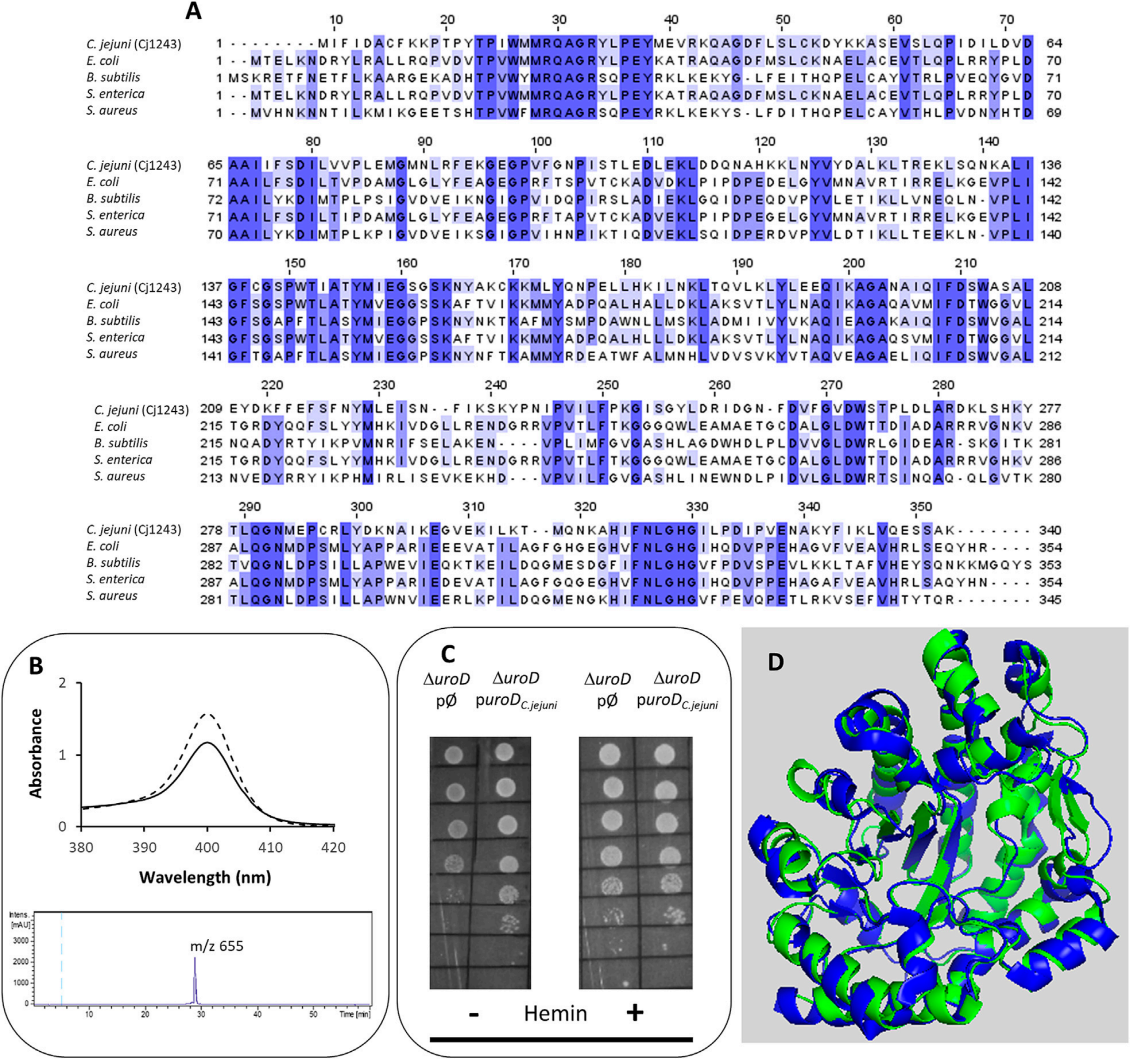
Cj1243 is a uro’gen III decarboxylase. **(A)** Amino acid sequence alignment of *C. jejuni* UroD (Cj1243, UniProt (UP): A8FMU8) and UroD from *E. coli* (UP: P29680), *B. subtilis* (UP: P32395), *Salmonella enterica* (NCBI-Protein ID: AGX12552), and *S. aureus* (UP: A6QI15), using Clustal Omega and edited using Jalview 2.11.2.5 (Waterhouse et al., 2009). Dark blue boxes highlight residues with a percentage identity >80%. The lighter the blue boxes, the lower the percentage identity of the highlighted residues (from 60% to 0%). **(B)** Top-image: UV-visible spectra of the coproporphyrin III standard (dashed line) and the oxidized form of the reaction product of *C. jejuni* UroD, i.e., coproporphyrin III (filled line). Lower image: the HPLC-MS extracted-ion chromatogram of the reaction product of the *C. jejuni* UroD reaction. **(C)** *E. coli* Δ*uroD* strain containing the empty plasmid (pØ) or a plasmid harboring the *cj1243* gene (p*uroD*_*C.jejuni*_) was diluted 1:10 in six steps and spotted on LB agar medium in the presence and absence of hemin (10 µM). **(D)** Structure model of *C. jejuni* UroD (green), obtained by AlphaFold2, superimposed with *B. subtilis* UroD structure (blue, PDB number 2INF), with a RMSD between the Cα carbons of 1.1.

The first conversion of uro’gen III involves the decarboxylation of the acetic acid groups of uro’gen III, promoted by UroD, yielding coproporphyrinogen III (copro’gen III). In the *C. jejuni* genome, the *cj1243* gene encodes a protein that shares amino acid sequence identity of approximately 45% and 38% with UroD of *E. coli* and *Bacillus* (*B.*) *subtilis*, respectively (Figure 2A).

The Cj1243 protein was produced recombinantly, purified, and tested for the conversion of uro’gen III to copro’gen III. We obtained the uro’gen III substrate from porphobilinogen using *D. vulgaris* PbgS and UroS enzymes, as described in the Materials and Methods section. The products of the reaction mixture containing *C. jejuni* Cj1243 and uro’gen III were analyzed by UV-visible spectroscopy and HPLC-MS (Figure 2B). The two typical features of the oxidized form of coproporphyrin III (copro’gen III), namely, the development of an absorbance at 400 nm in the visible spectrum and the presence of a mass peak at 655 m/z in the MS, confirmed the formation of copro’gen III.

We determined the *in vivo* activity of *C. jejuni* Cj1243 in a complementation assay using the *E. coli ΔuroD* mutant strain transformed with a plasmid that expresses *C. jejuni* UroD. The absence of the *uroD* gene in the *E. coli ΔuroD* strain prevents heme synthesis, so that the strain only grows in heme-supplemented medium (Săsărman et al., 1975). Figure 2C and Supplementary Figure S1 show that the expression of *C. jejuni* Cj1243 in *E. coli ΔuroD*, in the absence of any external source of heme, rescued the growth defect of *E. coli ΔuroD*, i.e., it restored the heme biosynthesis capacity of the mutant strain.

The predicted structure of Cj1243 obtained using AlphaFold2 (Figure 2D) displays a high degree of confidence, and the protein shares significant structural similarity with bacterial homologs. In particular, the predicted structure of Cj1243 aligns with that of *B. subtilis* UroD with a root mean square deviation (RMSD) of 1.1 and retains the main structural features of the *Bacillus* protein. Taken together, these results prove that Cj1243 is an active uro’gen decarboxylase UroD in *C. jejuni*.

### 3.2 Copro’gen III to proto’gen IX

Conversion of copro’gen III to protoporphyrinogen IX (proto’gen IX) requires oxidative decarboxylation of the propionate groups of rings A and B of copro’gen III to yield the vinyl groups of proto’gen IX. In bacteria, this reaction can be catalyzed by two distinct enzymes, namely, the aerobic manganese-containing copro’gen III decarboxylase (CgdC) and the anaerobic coproporphyrinogen dehydrogenase CgdH (Dailey et al., 2017; Cheng et al., 2022). BLAST searches of the *C. jejuni* genome did not identify genes encoding bacterial homologues of CgdC but revealed the presence of three CgdH-like proteins encoded by *cj0992c*, *cj0363c*, and *cj0580c*.

**FIGURE 3:**
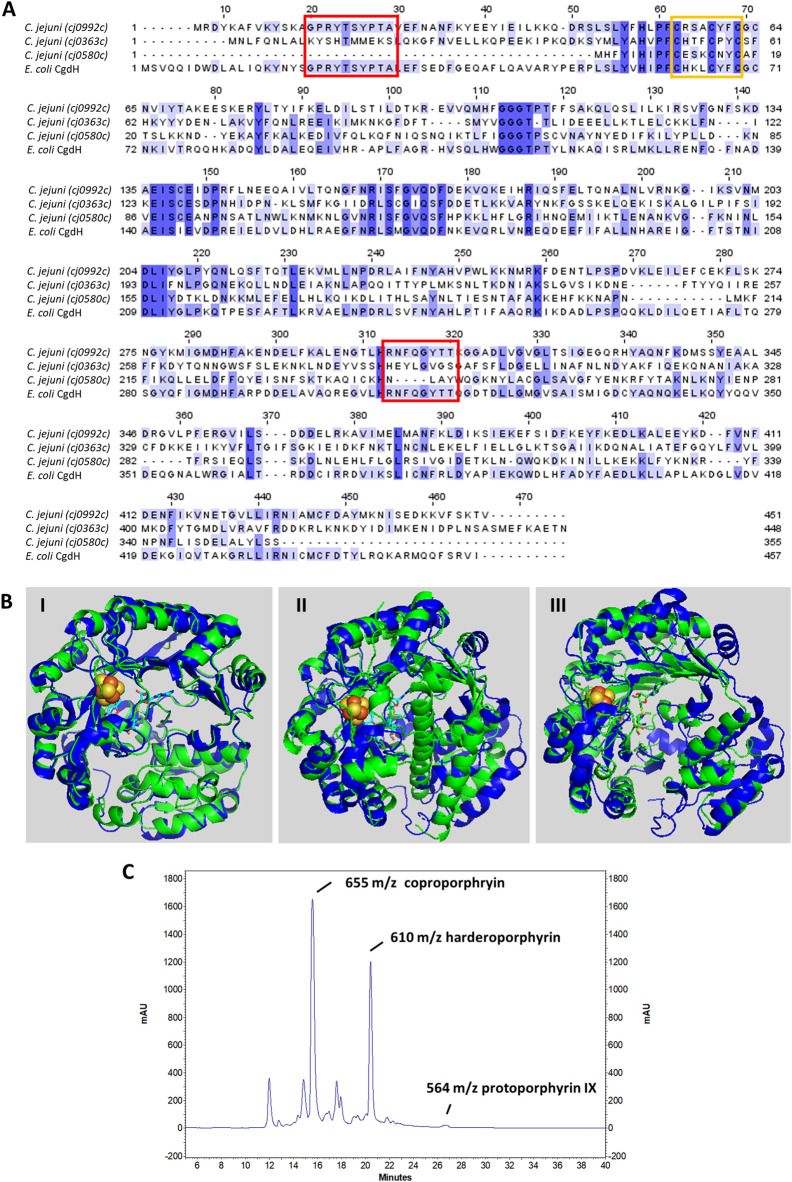
*C. jejuni* CgdH1 has copro’gen dehydrogenase activity. **(A)** Amino acid sequence alignment of *C. jejuni* CgdHs, namely, CgdH1 [Cj0992c, UniProt (UP): Q0P9R0], CgdH2 (Cj0363c, UP: Q0PBE7), CgdH3 (Cj0580c, UP: Q0PAT7), and CgdH from *E. coli* (UP: P32131), obtained using Clustal Omega and edited using Jalview 2.11.2.5. Dark blue boxes highlight residues with a percentage identity >80%. The lighter the blue boxes, the lower the percentage identity of the highlighted residues (from 60% to 0%). Red and orange boxes indicate the motifs required for coproporphyrinogen III dehydrogenase activity (GPRYTSYPTA and RNFQGYTT) and the CxxxCxxC sequence predicted to bind the Fe–S cluster, respectively. **(B)** Structures of *C. jejuni* proteins (green), namely, CgdH1 (Cj0992c) (I), CgdH2 (Cj0363c) (II), and CgdH3 (Cj0580c) (III), modelled by AlphaFold2 and aligned with *E. coli* CgdH (blue, PDB number 1OLT), with RMSD values of 0.8, 3.3, and 1.7, respectively. **(C)** HPLC chromatogram of the protoporphyrinogen IX reaction product of *C. jejuni* CgdH1 (Cj0992c). Peaks at m/z 564, 610, and 655 correspond to protoporphyrin IX (oxidized form of proto’gen IX), the intermediate harderoporphyrin I, and the oxidized form of the coproporphyrin III substrate, respectively. The control reaction is shown in Supplementary Figure S3.

*C. jejuni* Cj0992c and Cj0363c have a similar number of amino acid residues, specifically 451 and 448 amino acid residues, respectively, while Cj0580c is much shorter with only 355 amino acid residues. *C. jejuni* Cj0992 shares the highest similarity with *E. coli* CgdH (45% identity, 63% similarity, and 98% coverage). *C. jejuni* Cj0363c and Cj0580c share lower similarity with *E. coli* CgdH (26%–29% identity, 41%–48% similarity, ∼40% coverage) (Figure 3A). The *E. coli* CgdH motifs for coproporphyrinogen dehydrogenase activity (Layer et al., 2006; Dailey et al., 2015; Ji et al., 2019) are only present in Cj0992c (_14_GPRYTSYPTA_23_ and _311_HRNFQGYTT_319_).

Both Cj0992c and Cj0363c contain putative binding sites for S-adenosyl-L-methionine (SAM) and an Fe–S cluster. Cj0992c and Cj0363c possess a typical Radical SAM [4Fe–4S] binding motif (CX_3_CX_2_CXC) (Figure 3A).

The structures of *C. jejuni* Cj0992c, Cj0363c, and Cj0580c were predicted using AlphaFold2 and superimposed with that of *E. coli* CgdH (HemN) (Layer et al., 2003). The RMSD values for the superimposition were 0.8, 3.3, and 1.7, respectively. The structure of *C. jejuni* Cj0992c is predicted to retain the features of *E. coli* CgdH, including an N-terminal domain that is significantly larger than the C-terminal domain, which consists of three almost parallel α-helices (Figure 3B-I). The predicted structure of *C. jejuni* Cj0363c is quite different from that of *E. coli* CgdH [HemN; (Layer et al., 2003)], particularly due to the presence of an α-helix located between the N-terminal and C-terminal domains (Figure 3B-II). Although the modelled structure of *C. jejuni* Cj0580c appears more similar to the *E. coli* CgdH structure than to *C. jejuni* Cj0363c, the alignment of *C. jejuni* Cj0580c with Cj0363c has an RMSD of 1.7, indicating significant structural differences between the two proteins (Figure 3B-III).

The three *C. jejuni cgdH* genes are spread throughout the genome. The *cj0992c* gene is part of a gene cluster that also encodes a ferredoxin-type electron transport protein (Cj0991c), the hypothetical protein Cj0993c, an ornithine carbamoyltransferase ArgF (Cj0994c), and the delta-aminolevulinic acid dehydratase HemB/PbgS (Cj0995c). Genes *cj0363c* and *cj0580c* are scattered throughout the genome and not located near any recognizable heme-biosynthesis-related genes.

For the sake of simplicity, the three CgdH-like proteins encoded by *cj0992c*, *cj0363c*, and *cj0580c* will be designated as CgdH1, CgdH2, and CgdH3, respectively, from now on. We successfully cloned, expressed, and purified CgdH1 and CgdH2. However, despite several attempts, the low solubility of CgdH3 (Cj0580c) did not yield enough quantity for subsequent studies. Although CgdH1–2 contain Fe–S binding motifs, the proteins were isolated in the apo-form. Hence, prior to enzyme assays, *C. jejuni* CgdH1 and CgdH2 were treated to promote the incorporation of Fe–S clusters through reconstitution reactions carried out under anaerobic conditions (see Materials and Methods). After the reconstitution assays, CgdH1 and CgdH2 exhibited broad absorption bands typical of the presence of Fe–S centers (∼320 nm and 420 nm, respectively) (Supplementary Figure S2).

The copro’gen III dehydrogenase activity of the reconstituted CgdH1 and CgdH2 was evaluated in separate reactions. Each protein was incubated with copro’gen III, SAM, NADH, and cell extracts, using the conditions described in Materials and Methods, and the products were analyzed by HPLC-MS. The expected final reactional product, protoporphyrin IX (the oxidized form of proto’gen IX), was observed, although the reaction is incomplete as peaks corresponding to the substrate and the harderoporphyrin intermediate were present (Figure 3C). Similar activity assays conducted for *E. coli* HemN, and analyzed by MS, also generated several porphyrin products (Rand et al., 2010; Ji et al., 2019).

Although the same type of assay was performed with the reconstituted CgdH2, no formation of protoporphyrin IX or any other intermediate was observed. In other words, CgdH2 does not exhibit copro’gen III dehydrogenase activity.

### 3.3 Proto’gen IX to protoporphyrin IX

**FIGURE 4:**
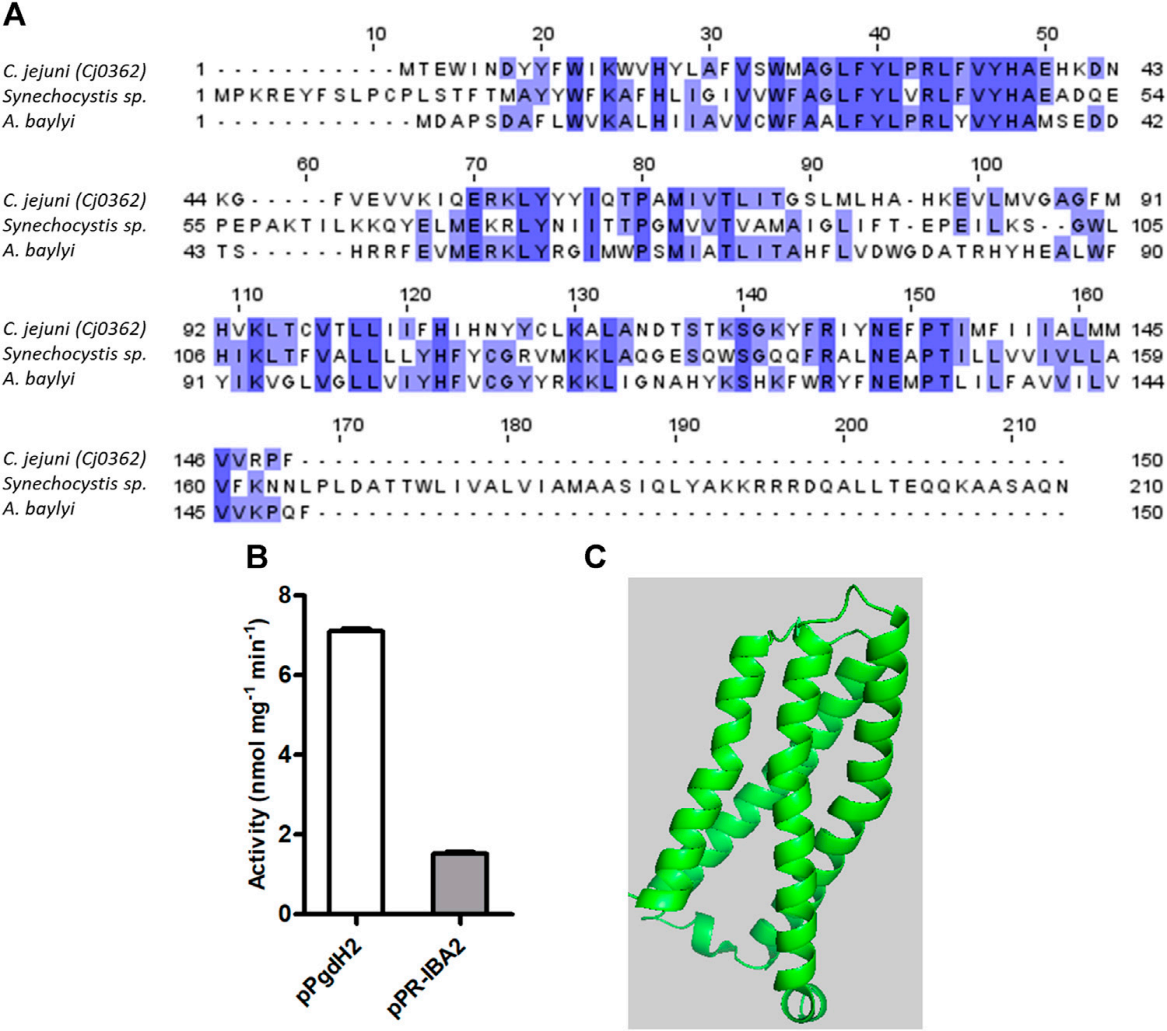
*C. jejuni* contains a proto’gen IX dehydrogenase enzyme. **(A)** Amino acid sequence alignment of *C. jejuni* PgdH2 [Cj0362, UniProt (UP): Q0PBE8], with two previously studied protoporphyrinogen oxidases, PgoX from *Synechocystis* sp. (UP: P72793) and *Acinetobacter baylyi*, (UP: Q6FDT1), carried out using Clustal Omega and edited using Jalview 2.11.2.5. Dark blue boxes highlight residues with a percentage identity >80%. The lighter the blue boxes, the lower the percentage of identity of the highlighted residues (from 60% to 0%). **(B)** Proto’gen IX conversion to protoporphyrin IX was assayed in *E. coli* cell membranes carrying the plasmid expressing *pgdH2* (pPgdH2) or the empty plasmid (pPR-IBA2). The reaction fluorescence of the mixture containing 500 nM proto’gen IX, 150 mM NaCl, 20% glycerol, 5 mM DTT, and 0.3 mg of membrane fraction of each *E. coli* cell extract was monitored for 20 min. The fluorescence intensity units were converted to concentration by interpolation of a calibration curve obtained with protoporphyrin IX standards. **(C)** Structure of *C. jejuni* PgdH2 (Cj0362) predicted by AlphaFold2.

Three different bacterial enzymes may perform the conversion of proto’gen IX to protoporphyrin IX, namely, PgoX, PgdH1, and PgdH2. A search of the *C. jejuni* genome retrieved a PgdH2-like enzyme (Cj0362) that exhibits 39% and 38% identity with PgdH2 from *Synechocystis* sp. and *Acinetobacter baylyi*, respectively (Figure 4A). Despite our efforts to purify *C. jejuni* Cj0362 to homogeneity, it was not possible to obtain enough quantities of the enzyme for the biochemical studies. Alternatively, the proto’gen IX oxidase activity was tested in the membrane fraction of *E. coli* cells containing Cj0362 and incubated with the proto’gen IX substrate. Cells containing Cj0362 exhibited much higher activity for the oxidation of proto’gen IX than cells without the protein (Figure 4B). This result indicates that Cj0362 is an active PgdH2 of *C. jejuni*. Since no structures are available for bacterial PgdH2-like proteins, we used AlphaFold2 to model Cj0362, which showed that the predicted structure is of the 4-helix-bundled type (Figure 4C).

### 3.4 Protoporphyrin IX to heme

**FIGURE 5:**
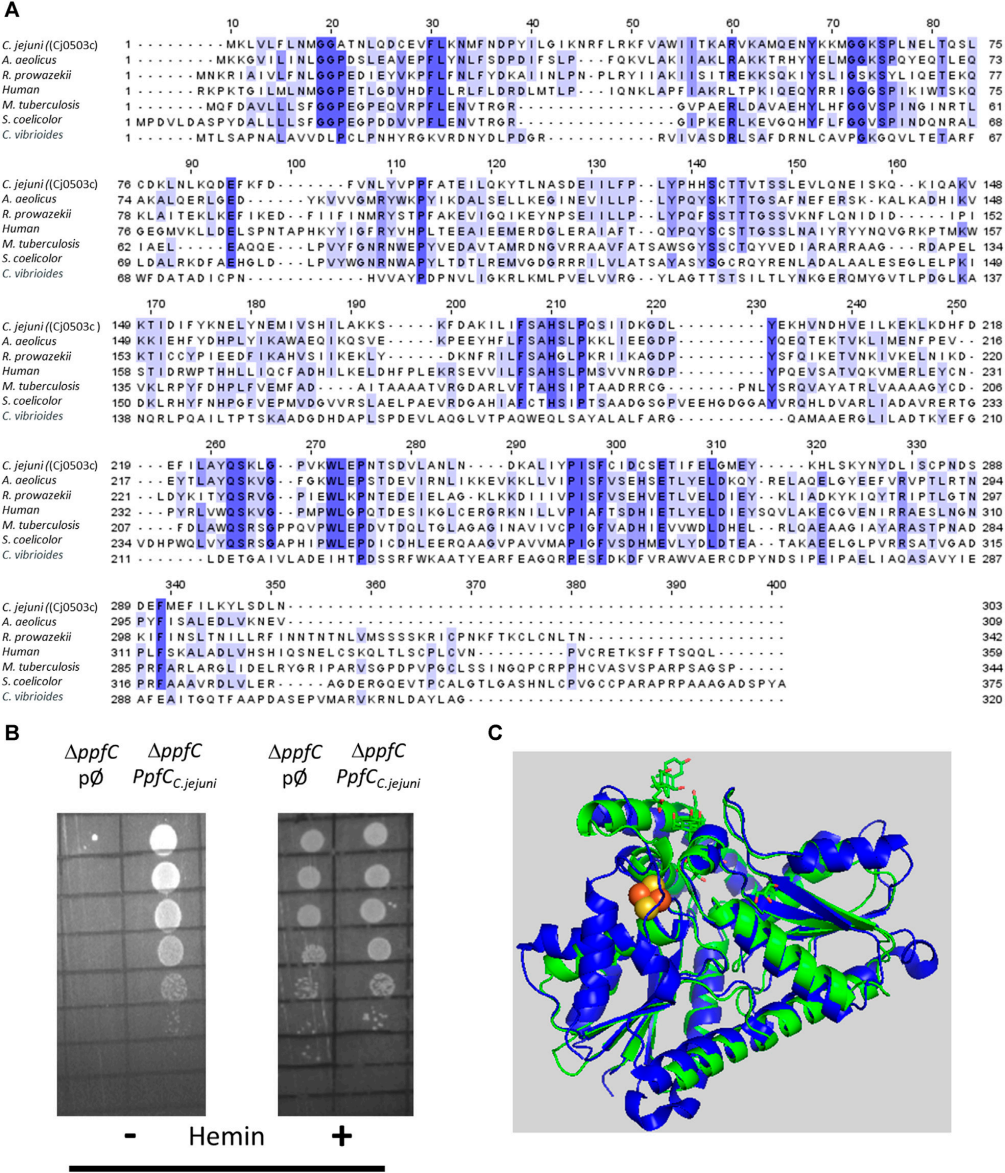
*Campylobacter jejuni* encodes a functional protoporphyrin ferrochelatase (PpfC). **(A)** Amino acid sequence alignment of *C. jejuni* PpfC [Cj0503c, UniProt (UP): Q9PI08], and PpfC from *A. aeolicus* (UP: O67083), *R. prowazek*ii (UP: Q9ZC84), Human (UP: P22830), *M. tuberculosis* (UP: P9WNE3), *S. coelicolor* (UP: O50533), and *C. vibrioides* (UP: Q9A3G2), carried out using Clustal Omega and edited using Jalview 2.11.2.5. Dark blue boxes highlight residues with a percentage identity >80%. The lighter the blue boxes, the lower the percentage identity of the highlighted residues (from 60% to 0%). **(B)** *E. coli* Δ*ppfC* strain transformed with the empty plasmid (pØ) or with a plasmid harboring the *C. jejuni cj0503c* gene (*ppfC*_*C.jejuni*_) was diluted 1:10 in six steps and spotted on LB agar medium in the absence (−) and in the presence (+) of hemin (10 µM). **(C)** Structure modelling using AlphaFold2 of *C. jejuni* PpfC (green) superimposed with that of human PpfC enzyme (blue, PDB number 1HRK), with an RMSD value of 1.2. Human PpfC enzyme binds a 2Fe–2S cluster that is represented with two yellow (Fe) and two red (S) spheres.

*C. jejuni* encodes Cj0503c that shares amino acid sequence identity between 22% and 37% with ferrochelatase of *Aquifex (A.) aeolicus*, *Rickettsia (R.) prowazekii*, Human, *Mycobacterium (M.) tuberculosis*, *Streptomyces (S.) coelicolor*, and *Caulobacter (C.) vibrioides* (Figure 5A). Therefore, the recombinant Cj0503c was produced, and the activity of the purified protein was evaluated. *C. jejuni* Cj0503c exhibits a ferrochelatase specific activity of 12.0 ± 1.3 nmol mg^−1^·min^−1^, a value that is in the same order of magnitude as that of other bacterial ferrochelatases, albeit these are coproporphyrin ferrochelatases (Hansson and Hederstedt, 1994; Lobo et al., 2015). Cj0503c activity was also assessed in the heme auxotrophic strain *E. coli* Δ*ppfC* mutant (Nakahigashi et al., 1991; Miyamoto et al., 1992). Complementation of *E. coli* Δ*ppfC* with *cj0503c* allowed growth in heme-free medium, showing that Cj0503c is a functional protoporphyrin ferrochelatase (Figure 5B; Supplementary Figure S1A).

The structure of Cj0503c, predicted by AlphaFold2, is depicted in Figure 5C. Although superimposition of Cj0503c with *B. subtilis* CpfC has a high RMSD of 2.9, the two structures share a similar overall structure and number of helices and beta sheets.

### 3.5 *C. jejuni* CgdH2 is a heme-binding protein

**FIGURE 6:**
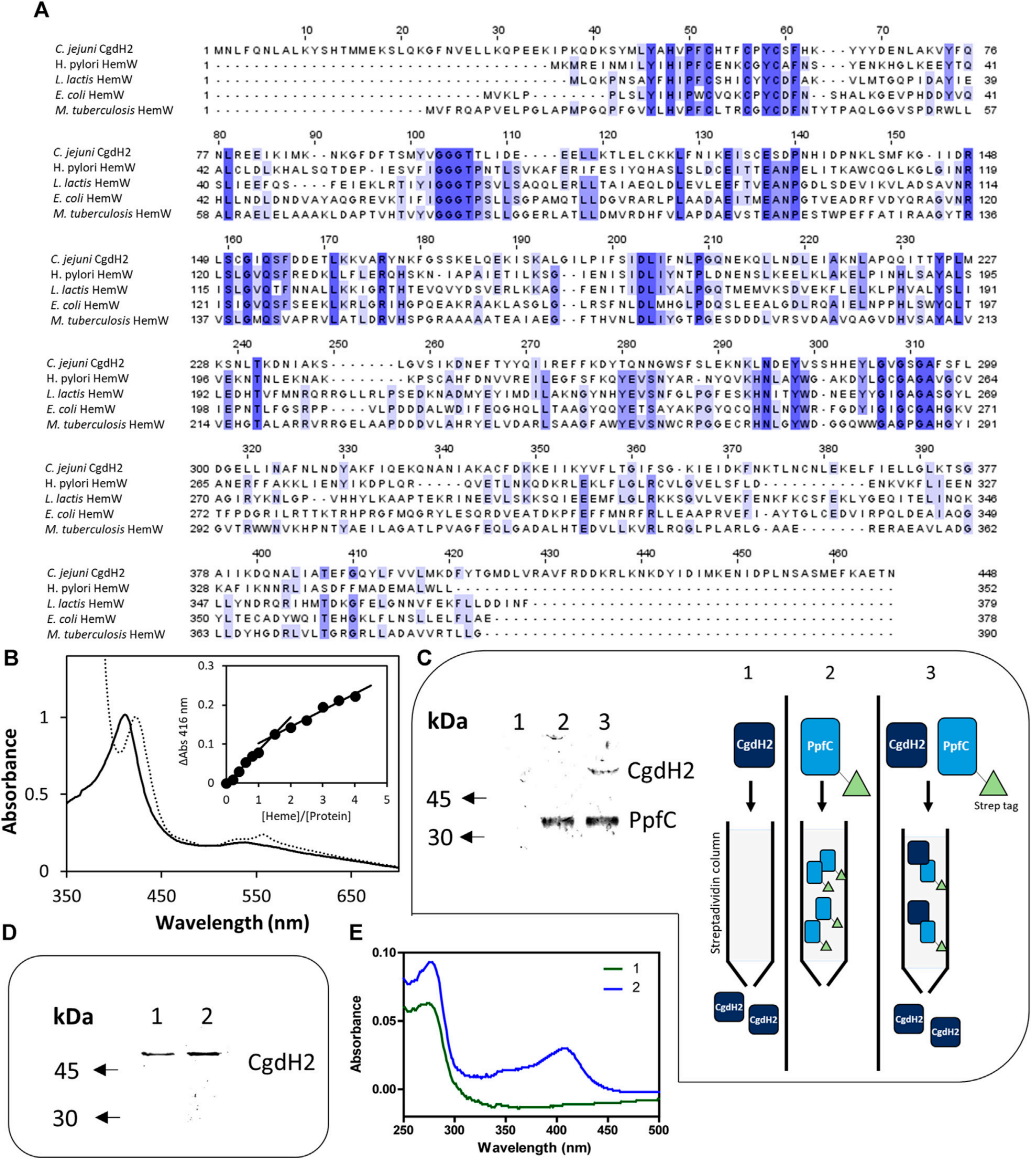
CgdH2 is a heme-binding protein that receives heme from ferrochelatase PpfC. **(A)** Amino acid sequence alignment of *C. jejuni* CgdH2 [Cj0363c, UniProt (UP): Q0PBE7], and HemW from *Helicobacter (H.) pylori* (UP: O25824), *Lactococcus lactis* (UP: Q9CGF7), *E. coli* (UP: P52062), and *M. tuberculosis* (UP: P9WP73), carried out using Clustal Omega and edited using Jalview 2.11.2.5. Dark blue boxes highlight residues with a percentage identity >80%. The lighter the blue boxes, the lower the percentage identity of the highlighted residues (from 60% to 0%). **(B)** Spectroscopic analysis of heme-loaded CgdH2 before (^____^) and after reduction with dithionite (----). Inset depicts the titration assay of CgdH2 with increasing concentrations of hemin. ΔAbs represent the absorbance at 416 nm subtracted from the absorbance of the buffer containing only hemin in the corresponding concentration. **(C)** Left, pull-down assay. Apo-PpfC-Strep, apo-CgdH2-His, and the mixture of the two apo-proteins were loaded, separately, onto a Strep-Tactin Sepharose column. Apo-CgdH2-His does not bind to the column and is eluted with the washing buffer A (Tris-HCl 50 mM pH 8). Right, schematic representation of the pull-down assay. 1) Apo-CgdH2 does not bind to Strep-tactin resin. 2) Apo-PpfC-Strep is eluted with 5 mM desthiobiotin. 3) When both proteins are loaded into the Strep-tactin column, apo-CgdH2 is retained due to the interaction with apo-PpfC. Fractions eluted with buffer A containing 5 mM desthiobiotin were analyzed by SDS-PAGE: lane 1- apo-CgdH2; lane 2- mixture of apo-PpfC-Strep with apo-CgdH2; and lane 3- apo-PpfC-Strep. **(D,E)** Heme transfer assay. Fractions collected after loading apo-CgdH2 onto an empty resin 1) and onto a resin previously bound to the heme-ferrochelatase (He-PpfC-Strep) 2) and eluted with buffer B were analyzed by SDS-PAGE (E) and UV-visible spectra (F).

We observed that *C. jejuni* CgdH2 lacks coproporphyrinogen dehydrogenase activity. A more extensive BLAST analysis revealed that CgdH2 shares some degree of similarity with the heme chaperon HemW (e.g., *E. coli* HemW: 24% identity, 49% similarity, 71% coverage) (Figure 6A). This similarity led to the hypothesis that CgdH2 could be a heme-binding protein, which was tested in two types of assays.

In the first assay, *E. coli* cell extracts containing CgdH2 were pre-incubated with hemin before conducting the affinity purification of CgdH2, as described in Materials and Methods. The purified CgdH2 contained approximately one heme molecule (0.8 ± 0.1 heme/protein), as determined by the pyridine hemochromagen assay.

In the second assay, CgdH2 was isolated after production in *E. coli*, and the ability of CgdH2 to bind heme was tested by titrating purified apo-CgdH2 with hemin. The addition of increasing concentrations of hemin to apo-CgdH2 led to an increase in the Soret band at 416 nm (Figure 6B). Data analysis showed that CgdH2 binds one heme molecule per protein, consistent with the result obtained in the aforementioned assay. The K_d_ for heme binding of CgdH2 is 4.9 ± 1.0 µM, a value that is within the range of the values described for other heme chaperones, such as K_d_∼8 μM for *Lactococcus lacti*s HemW (Abicht et al., 2012), K_d_ of ∼4.3 μM for *C. jejuni* Cj1386 (Flint and Stintzi, 2015), and K_d_ of 0.2 μM for *Pseudomonas aeruginosa* PhuS heme trafficking protein (Bhakta and Wilks, 2006).

We also tested whether CgdH2 harboring the Fe–S cluster would bind heme. To carry out this, the Fe–S-reconstitued CgdH2 protein was incubated with 100 μM of hemin and passed through a gel filtration column. The spectrum showed that CgdH2 contains both cofactors, indicating that the presence of the Fe–S center does not prevent heme binding (Supplementary Figure S2B).

Since we observed that the fully loaded CgdH2 has features resembling a low-spin heme (Figure 6B), we next sought to identify the heme-binding ligands by site-directed mutagenesis. We selected amino acids that often serve as heme ligands, i.e., histidine, tyrosine, and methionine. The following residues were modified: histidines H48, H53, H62, H133, and H285; tyrosines Y46, Y66, and Y224, and methionines M44 and M227. The proteins were produced in *E. coli*, and cell extracts with each of the Cgdh2 mutant variants were prepared and incubated separately with hemin, as described previously. Following the purification step, the heme content of the purified proteins was determined by using the pyridine hemochromagen method. Based on the data shown in Table 1, amino acid residues H48 and H133 are predicted to be the heme ligands in CgdH2.

**TABLE 1:** CgdH2 binds heme. Heme content of CgdH2 wild-type and mutant proteins following incubation with hemin and purification and quantified by the pyridine hemochromagen assay. Results are expressed as the ratio of heme per protein.

CgdH2 protein	Heme/protein	HemW^a^
Wild type	0.8 ± 0.1	—
**H48**L	0.3 ± 0.0	yes
H53L	0.7 ± 0.0	no
H62L	0.7 ± 0.0	no
**H133**L	0.4 ± 0.0	no
H285L	0.8 ± 0.2	yes
Y46L	0.7 ± 0.1	yes
Y66L	0.7 ± 0.1	no
Y224L	0.8 ± 0.1	yes
M44L	0.8 ± 0.1	no
M227L	0.8 ± 0.2	no

^a^ Presence of equivalent residues in bacterial HemW proteins, as depicted in Figure 6A; heme ligands of CgdH2 are highlighted in bold.

### 3.6 *C. jejuni* CgdH2 receives heme from ferrochelatase

In bacteria, it is not known how heme is transferred from ferrochelatase to the target proteins. The heme-binding capability of *C. jejuni* CgdH2 prompted us to investigate whether the protein could function as an intracellular heme receptor. Additionally, we considered a potential connection with enzymes involved in heme biogenesis, particularly the ferrochelatase PpfC, which in *C. jejuni* is the final enzyme in the PPD pathway. To explore this, we constructed, expressed, and produced two *C. jejuni* apo-proteins: CgdH2 linked at the C-terminus to His-tag (apo-CgdH2-His) and PpfC fused at the N-terminus to Strep-tag (apo-PpfC-Strep). The interaction between the two proteins was analyzed with a pull-down assay (Figure 6C). Apo-PpfC-Strep, apo-CgdH2-His, and a mixture of the two were loaded separately onto a Strep-Tactin Sepharose column, which selectively binds Strep-tagged proteins. As shown in Figure 6C, apo-CgdH2-His did not bind to the resin, and apo-PpfC-Strep was eluted by the addition of 5 mM desthiobiotin. However, when the two apo-proteins, CgdH2 and PpfC, were pre-incubated and the mixture was loaded onto the resin, CgdH2 was retained and eluted together with PpfC, indicating their interaction.

To test the transfer of heme between the two proteins, we prepared He-PpfC (heme-containing PpfC) and confirmed the tight binding of heme to PpfC. We observed that no free heme was spontaneously released from the column during the washing buffer steps, and the He-PpfC that was eluted with 5 mM desthiobiotin still retained ∼1 heme/protein.

To investigate the heme transfer from He-PpfC to CgdH2, we conducted the following experiment: we loaded apo-CgdH2 onto a Strep-Tactin Sepharose resin that has been pre-bound with He-PpfC-Strep. To overcome the experimental limitation of distinguishing which protein had bound heme when CgdH2 is eluted together with He-PpfC, we used an excess of apo-CgdH2 relative to the concentration of He-PpfC bound to the resin (2:1). As a result, we observed that the part of CgdH2 that was eluted with the washing buffer contained bound heme (∼0.4 heme per protein) (Figure 6E). This finding strongly supports the conclusion that heme transfer from He-PpfC to CgdH2 takes place. Thus, CgdH2 can be identified as a novel heme-binding protein.

**FIGURE 7:**
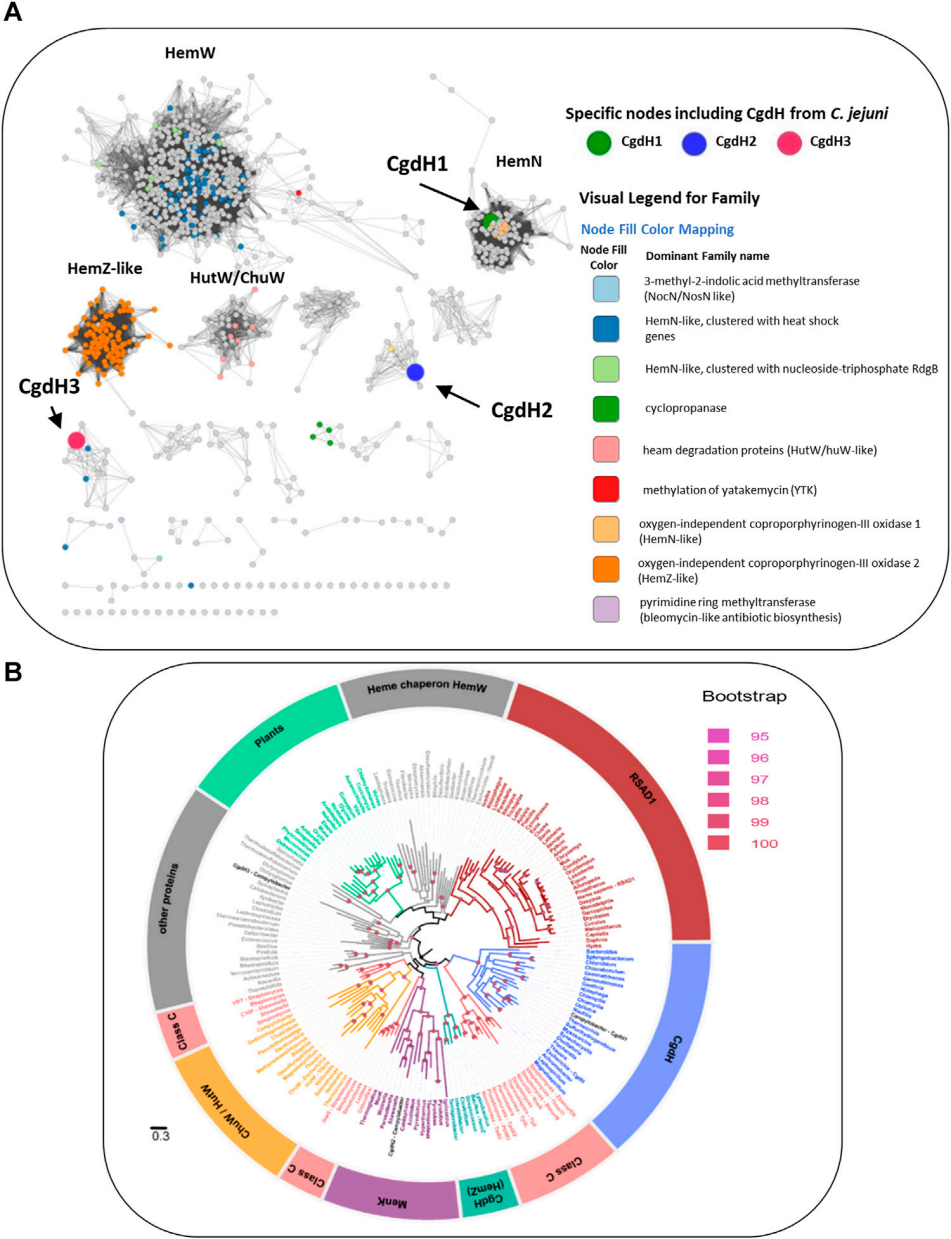
Network distribution of the radical S-adenosyl-L-methionine (SAM) CgdH-like enzymes. **(A)** Network distribution obtained using the Structure Function Linkage Database (SFLD). Nodes containing CgdH1, CgdH2, and CgdH3 are represented by large green, blue, and red dots, respectively. CgdH1 is included in the bacterial HemNs cluster, while CgdH2 and CgdH3 are located in clusters containing proteins of unknown function. Of note, *Gordonibacter* MenK (CBL03906.1; Uniprot: D6E859) clusters with *C. jejuni* CgdH2 (blue dot) and is represented as a yellow dot. **(B)** Maximum likelihood phylogenetic reconstruction of CgdH-like proteins. The phylogeny was rooted using the minimal ancestor deviation method. Ultrafast bootstrap values above 95 (significant) are indicated by filled circles. The different clades are colored based on the functionally characterized CgdH-like protein that is present. The clades obtained were named as: Heme chaperone HemWs, RSAD1 (Radical S-adenosylmethionine domain-containing 1), CgdH (*bona fide* CgdHs), Class C (class C of Radical SAM methylases), CgdH (HemZ), MenK (menaquinones methyltransferases), ChuW/HutW (heme-degrading enzymes), other proteins (other HemN like proteins), and plants (unidentified CgdH-like proteins from plants) (Supplementary Table S4). The genus of the organism containing the CgdH-like protein sequence is indicated. The scale bar indicates the number of substitutions per site.

We conducted a cluster analysis for the three CgdH proteins using the Structure Function Linkage Database (SFLD) (Akiva et al., 2014) of the Radical SAM (RSM) proteins superfamily, which is a subgroup of the coproporphyrinogen III oxidase-like proteins. The results showed that CgdH-like proteins can be divided into nine functional types, including the canonical HemN enzymes, HemZ-like, HemW, and ChuW/HutW (Figure 7A). *C. jejuni* CgdH1 is grouped with *bona fide* HemN enzymes, in agreement with our experimental data. The node contains *C. jejuni* CgdH2 (represented by a large blue dot in Figure 7A) clusters with Gordonibacter MenK (UniProt: D6E859; yellow dot in Figure 7A) and other unidentified CgdH-like proteins. Importantly, this clade is not adjacent to the cluster containing HemW proteins, clearly showing that CgdH2 belongs to a distinct protein family (Figure 7A).

The phylogenetic study of the *C. jejuni* CgdH proteins was conducted considering the class of CgdH-like proteins, formerly designated as HemN, which includes anaerobic coproporphyrinogen III oxidases, methyltransferases, cyclopropanases, and heme chaperones, according to the classification presented in Cheng et al., 2022. A total of 177 sequences from 168 species were used for the phylogenetic reconstruction, resulting in the retrieval of 11 clades corresponding to characterized enzymes (Figure 7B). *C. jejuni* CgdH1 clustered with the CgdH *bona fide* branch, whereas *C. jejuni* CgdH3 branches in a clade containing other proteins of unknown function. Notably, *C. jejuni* CgdH2 did not fall within these clades or the branch of heme chaperon HemW proteins. Instead, CgdH2 appeared in the branch of menaquinone methyltransferases MenK, which includes several archaeal proteins. However, the *C. jejuni* CgdH2 protein is within a subclade of bacterial MenK sequences where other proteobacterial organisms are also included (Figure 7B).

Menaquinone methyltransferases, such as MenK/MenK2/MqnK, belong to the HemN-like class C radical SAM methyltransferases (class C RSMTs), which share a similar structure consisting of an N-terminal catalytic domain, a linker domain, and a C-terminal HemN domain. The analysis of a large number of protein sequences, namely, twelve seed class C RSMTs and ten other characterized radical SAM proteins that were grouped in eight main clusters designated as HemW, HemZ, ChuW, HutW, MqnK/MenK/MenK2 (MKMT), Jaw5, NosN, and C10P (Wilkens et al., 2021), identified six signature motifs, and their occurrence allowed defining the HemW and MKMT subgroups (Wilkens et al., 2021). We analyzed the presence of these motifs in CgdH2 when compared with those in HemW and MKMT proteins. Table 2 shows that the LYxHxPFCxxxCxxCxF motif, which ligates the [4Fe–4S] center, is conserved in all proteins. The two motifs, YFxxxRxE and QxTxYPLx, which are absent in HemWs, occur in CgdH2. Motifs GxxFxxxYxGGGT and NxFxxxxY, which have very low occurrences in HemW (∼2%), are present in CgdH2. Moreover, CgdH2 does not contain the H250NxxYW255 motif (*E. coli* HemW numbering) that is conserved in all bacterial HemW (Dailey et al., 2015) (Figure 3A). In addition, the superimposition of the predicted structures of *E. coli* HemW and CgdH2 shows a non-significant RMSD of 2.6.

**TABLE 2:** Occurrence of specific sequence motifs in CgdH2, menaquinone methyltransferases (MKMTs), and HemW-like chaperones.

	Motif sequence occurrence		
Sequence motif	CgdH2^a^	MKMT (828)^b^	HemW (7747)^b^
LYxHxPFCxxxCxxCxF	yes	96.0	29.4
YFxxxRxE	yes	91.7	0.1
GxxFxxxYxGGGT	yes	97.1	2.2
RxSxGxQxFxxxxL	yes	97.6	76.9
QxTxYPLx	yes	89.7	0.0
NxFxxxxY	yes	95.0	2.0

^a^ Presence of the sequence motif in CgdH2. Values represent the percentage of proteins containing the sequence motif relative to the total number of proteins present in the class.

^b^ As reported by Wilkens et al., 2021.

Therefore, we concluded that CgdH2 is not a HemW-like protein but constitutes a novel type of heme chaperone.

## 4 Discussion

**FIGURE 8:**
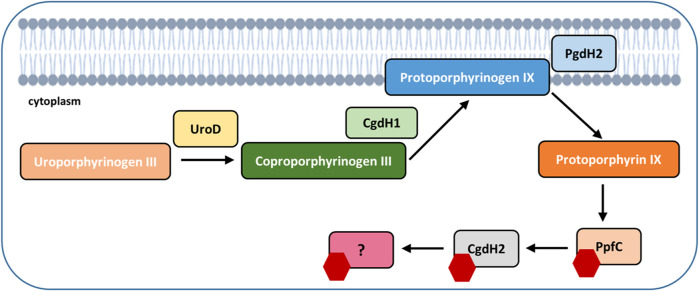
*C. jejuni* uses the protoporphyrin-dependent (PPD) pathway to form heme. Uroporphyrinogen III decarboxylase (UroD, Cj1243) catalyzes the decarboxylation of uroporphyrinogen III, yielding coproporphyrinogen III; oxygen-independent coproporphyrinogen III dehydrogenase (CgdH1, Cj0992c) catalyzes the oxidative decarboxylation of propionate substituents on pyrrole rings A and B of coproporphyrinogen III to the corresponding vinyl groups of protoporphyrinogen IX. In the penultimate step of the pathway, protoporphyrinogen IX is oxidized to protoporphyrin IX by protoporphyrinogen IX dehydrogenase (PgdH2, Cj0362), followed by insertion of ferrous iron into protoporphyrin IX, originating heme (red hexagon), a reaction that is catalyzed by protoporphyrin ferrochelatase (PpfC, Cj0503c). CgdH2 (Cj0363c) receives heme from ferrochelatase (PpfC, Cj0503c) and transfers heme to not yet known target proteins.

Bacterial heme biosynthesis begins with the formation of 5-aminolevulinic acid, which is converted to the intermediate tetrapyrrole macrocyclic uro’gen III. The biochemical characterization of several enzymes has allowed us to show that in the Gram-negative *C. jejuni* the transformation of uro’gen III into heme occurs through a PPD-like pathway (Figure 8). Furthermore, we have clarified which of the three putative copro’gen dehydrogenase proteins, retrieved by BLAST search analysis of the *C. jejuni* genome, is the enzyme acting in the heme biogenesis pathway.

The heme formed in ferrochelatase presumably needs to be escorted by protein chaperones to the final destination in a highly controlled manner. Several proteins may play this role based on their heme-binding affinity, such as the group of chaperone-like monoheme protein HemWs (Haskamp et al., 2018). *E. coli* HemW exhibits weak SAM cleavage activity and harbors a [4Fe–4S] that, although not required for heme binding, is necessary for heme transfer to the membrane subunit NarI of the respiratory nitrate reductase NarGHI. The transfer of heme from HemW to a catalytically inactive enzyme NarI restores nitrate reductase activity. *E. coli* HemW interacts with NarI and bacterioferritin but does not interact with catalase KatA or ferrochelatase (Haskamp et al., 2018).

Another type of heme chaperones are the mycobacterial orthologues, which were shown, by phylogenetic analysis, to form a divergent clade that includes the HemW proteins of other species such as *E. coli* and *L. lactis* (Sharma et al., 2021). Molecular dynamics studies have shown that in mycobacteria, HemW binds heme through the conserved H_250_NXXYW_255_ motif. This motif is not present in *C. jejuni* CgdH2; while H45 is highly conserved in all HemW-like proteins, residue H133 is unique to CgdH2 (Figure 5A). Moreover, mutant analysis indicated that histidine residues H45 and H133 are required to bind and/or stabilize the heme ligation in CgdH2.

Cj1386 is a heme-binding chaperon previously described in *C. jejuni*, which binds one heme coordinated through tyrosine Y57 (Flint and Stintzi, 2015). The decreased hemin content of KatA in the *Δcj1386* mutant was proposed to account for the reduced catalase activity and, consequently, the higher sensitivity of the strain to oxidative stress. Therefore, Cj1386 is considered a chaperon that binds and traffics heme to KatA (Flint and Stintzi, 2015). *C. jejuni* CgdH2 (447 aa) and Cj1386 (156 aa) share low sequence similarity (I <30%, query coverage of 18%). Furthermore, the *C. jejuni ΔcgdH2* mutant does not show modified sensitivity to oxidative stress compared to the wild-type strain, and CgdH2 does not interact with KatA (data not shown).

The interaction between ferrochelatases and other enzymes of the heme biosynthesis pathway has been previously reported as follows: in the cyanobacterium *Thermosynechococcus elongatus*, ferrochelatase interacts with proto’gen IX oxidase (Masoumi et al., 2008). In *Vibrio vulnificus*, a complex between ferrochelatase and proto’gen IX dehydrogenase was described (Kim et al., 2018). In *B. subtilis*, CpfC ferrochelatase co-purifies with Fra, a homolog of the eukaryotic iron chaperon frataxin (Mielcarek et al., 2015). In *S. aureus*, there is a transient interaction between ferrochelatase and coproheme decarboxylase ChdC (Celis et al., 2019). Additionally, in *S. aureus*, ferrochelatase CpfC interacts with heme oxygenase IsdG of the heme uptake system (Videira et al., 2018; Zamarreño Beas et al., 2022). In this work, we provide evidence for the interaction of ferrochelatase and the transfer of heme to a novel type of heme chaperone. *C. jejuni* CgdH2 belongs to the radical SAM superfamily, which includes the canonical CgdH-like enzymes, heme-degrading enzymes, and heme chaperones. However, CgdH2 falls outside of the HemW-containing clusters, as shown by the SFLD and phylogenetic analysis (Figure 7). Interestingly, CgdH2 appears in the clade of menaquinone methyltransferase-like proteins (MKMT) due to the conservation of several amino acid sequence motifs. The homology between the sequence and structural features of CgdH2 and MKMTs (e.g., RMSD of ∼0.89 between *C. jejuni* CgdH2 and *Gordonibacter pamelaeae* MenK) also raises the possibility of involvement of CgdH2 in methylation reactions, a hypothesis that needs to be addressed in future studies.

## 5 Conclusion

Altogether, our biochemical and phylogeny distribution data show that *C. jejuni* CgdH2 is a novel type of heme chaperone, with features that differ from those of bacterial HemWs. Moreover, our results show that CgdH2 can accept heme bound to the ferrochelatase enzyme from the PPD pathway.

The presence in *C. jejuni* of at least two heme chaperones also shows that heme homeostasis requires multiple chaperones with specific functions. The data presented herein expand our knowledge of the players involved in the intracellular binding and transportation of heme.

## Data Availability

The original contributions presented in the study are included in the article/Supplementary Material; further inquiries can be directed to the corresponding author.
