# Supplementary material for: In Campylobacter jejuni, a new type of chaperone receives heme from ferrochelatase
Source: Front Genet. 2023 Jun 21;14:1199357. doi: 10.3389/fgene.2023.1199357 (PMC10320005; doi:10.3389/fgene.2023.1199357)
Supplement: Supplementary file 1 [file DataSheet1.docx]

Supplementary Material

In *Campylobacter jejuni* a new type of chaperone receives heme from ferrochelatase

**Jordi Zamarreño Beas, Marco A.M. Videira, Val Karavaeva, Frederico S. Lourenço, Mafalda R. Almeida, Filipa Sousa, and Lígia M. Saraiva***

*** Correspondence:** Lígia M. Saraiva: lst@itqb.unl.pt

# Supplementary Figures

| **A** | **B** |
| --- | --- |

**Supplementary Figure 1. Functional complementation by *C. jejuni* UroD and PpfC enzymes**. **A.** *Escherichia coli* wild type, Δ*uroD*, aqnd Δ*ppfC* strains containing the empty vector (pØ) or plasmid encoding the corresponding *C. jejuni* homologous gene (P*uroD_C.jejuni_* or P*pfC_C.jejuni_*) streaked on LB-agar medium with no hemin. **B.** UV-Visible spectrum of the oxidized form of the reaction product of *C. jejuni* UroD.

| **A** | **B** | **C** |
| --- | --- | --- |
| **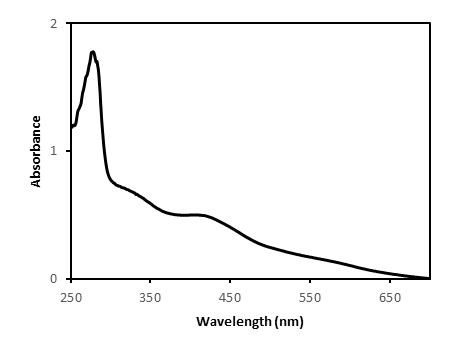** | 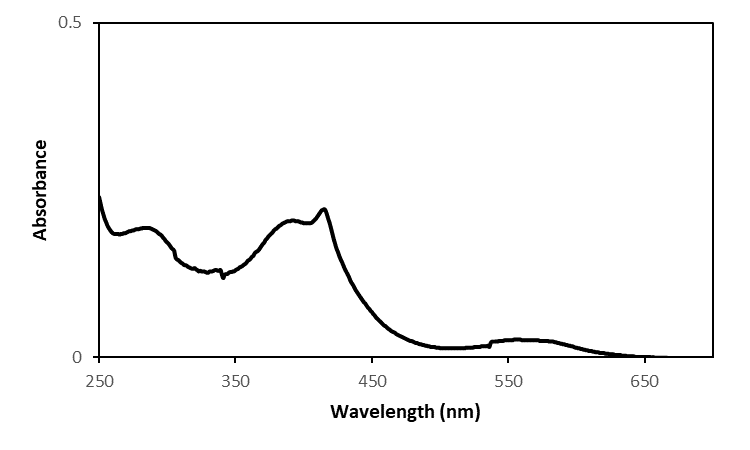 |  |

**Supplementary Figure 2. UV-Visible spectra of *C. jejuni* CgdH proteins.** **A**. CgdH1 (50 µM) spectrum exhibits two broad bands centered around 330 nm and 420 nm characteristic of a [4Fe-4S]^2+^ cluster. **B**. *C. jejuni* CgdH2 (20 µM) spectrum showing the presence of heme and Fe-S cofactors. **C.** *C. jejuni* CgdH2 containing only the Fe-S center.

**655 m/z coproporphyrin**

**Supplementary Figure 3. HPLC chromatogram of copro’gen III.** The product of the control reaction obtained in the absence of *C. jejuni* Cj0992c, with peak at m/z of 655 corresponding to coproporphyrin III (oxidized form of copro’gen III).

# Supplementary Tables

**Supplementary Table 1.** Strains used in this study.

| **Organism** | **Strain** | **Genotype** | **Resistance** | **Reference** |
| --- | --- | --- | --- | --- |
| *C. jejuni* | ATCC 700819  NCTC 1168 |  | - | NCTC collection* |
| *E. coli* | BL21(DE3) pLysS | F^-^*ompT* *hsdS*_B_(r_B_^-^ m_B_^-^) *gal dcm* (DE3) pLysS | Cm^R^ |  |
| *E. coli* | BW25113 |  | - | Baba et al 2006 |
| *E. coli* | IH71 | F^+^ Δ*visA*(=Δ*ppfC*) HfrC *lac*_am125_ *trp*_am_ *su*^O^ | - | NBRP-*E. coli* at NIG** |
| *E. coli* | IH81 | Δ*hemE(=*Δ*uroD*)*::cat* | Cm^R^ | NBRP-*E. coli* at NIG** |

* National Collection of Type Cultures collection

** National BioResource Project *E. coli* strain at National Institute of Genetics, Microbial Physiology Laboratory, 1111 Yata, Mishima, Shizuoka, 411-8540 Japan

Cm^R^ – chloramphenicol resistance (*cat* gene)

**Supplementary Table 2.** Primers used in this study.

| **Primer Name** | **Plasmid** | **Restriction sites** | **Gene** | **Sequence 5´-3 ´** |
| --- | --- | --- | --- | --- |
| Forward *uroD* | pPR-IBA 2 | KpnI | *uroD* | GGCCGGTACCCATGATTTTTATCGATGCTTGC |
| Reverse *uroD* | pPR-IBA 2 | AvrII, BamHI | *uroD* | CCGGGGATCCTTAACCTAGGTCATTTAGCTGAACTTTCTT |
| Forward *ppfC* | pPR-IBA 2 | EcoRI | *ppfC* | CCGGGAATTCGGTGAAATTAGTTTTATTTTT |
| Reverse *ppfC* | pPR-IBA 2 | AvrII, BamHI | *ppfC* | CCGGGGATCCTAACCTAGGTTAGTTAAGATCAGATAAAT |
| CgdH1_pET23b_Fw_NdeI | pET23b | NdeI | *cgdH1* | CCGGCATATGAGAGATTATAAAGCTTTTG |
| CgdH1_pET23b_Rv_XhoI | pET23b | XhoI | *cgdH1* | CCGGCTCGAGCACCGTTTTTGAGAATAC |
| CgdH2_pET23b_Fw_NdeI | pET23b | NdeI | *cgdH2* | CCGGCATATGAATTTATTTCAAAATTTAGC |
| CgdH2_pET23b_Rv_XhoI | pET23b | XhoI | *cgdH2* | CCGGCTCGAGGTTTGTCTCCGCTTTAAATT |
| CgdH3_pET23b_Fw_NdeI | pET23b | NdeI | *cgdH3* | CCGGCATATGCATTTTTATATCCATATTCC |
| CgdH3_pET23b_Rv_XhoI | pET23b | XhoI | *cgdH3* | CCGGCTCGAGAGATGAGAGATATAAAGCAA |

**Supplementary Table 3.** Plasmids used in this study.

| **Plasmid*** | **Plasmid description** |
| --- | --- |
| pPR-IBA2-*uroD* | *C. jejuni uroD* fused to Strep tag at N-terminus |
| pPR-IBA2-*ppfC* | *C. jejuni ppfC* fused to Strep tag at N-terminus |
| pPR-IBA2-*pgdH2* | *C. jejuni pgdH2* fused to Strep tag at N-terminus |
| pET-23b-*cgdH1* | *C. jejuni cgdH1* fused to Strep tag at N-terminus |
| pET-23b-*cgdH2* | *C. jejuni cgdH2* fused to His tag at C-terminus |
| pET-23b-*cgdH3* | *C. jejuni cgdH3* fused to His tag at C-terminus |
| pET-23b-cgdH2Y66L | *C. jejuni cgdH2*Y66L fused to His tag at C-terminus |
| pET-23b-*cgdH2*Y46L | *C. jejuni cgdH2*Y46L fused to His tag at C-terminus |
| pET-23b-*cgdH2*H48L | *C. jejuni cgdH2*H48L fused to His tag at C-terminus |
| pET-23b-*cgdH2*M44L | *C. jejuni cgdH2*M44L fused to His tag at C-terminus |
| pET-23b-*cgdH2*Y244L | *C. jejuni cgdH2*Y244L fused to His tag at C-terminus |
| pET-23b-*cgdH2*H62L | *C. jejuni cgdH2*H62L fused to His tag at C-terminus |
| pET-23b-*cgdH2*H53L | *C. jejuni cgdH2*H53L fused to His tag at C-terminus |
| pET-23b-*cgdH2*H285L | *C. jejuni cgdH2*H285L (fused to His tag at C-terminus |
| pET-23b-*cgdH2*H133L | *C. jejuni cgdH2*H133L fused to His tag at C-terminus |
| pET-23b-*cgdH2*M227L | *C. jejuni cgdH2*M227L fused to His tag at C-terminus |

*All plasmids mediate resistance to ampicillin.

**Supplementary Table 4.** List of proteins used for the phylogenetic tree analysis.

| CgdH type | Accession No. | Strain |
| --- | --- | --- |
| CgdH | AAN83245 | *Escherichia coli* |
| CgdH | WP_028069898 | *Sphingobacterium thalpophilum* |
| CgdH | WP_006365438 | *Chlorobium ferrooxidans* |
| CgdH | WP_069810592 | *Chlorobaculum limnaeum* |
| CgdH | WP_026853902 | *Geothrix fermentans* |
| CgdH | WP_005034428 | *Holophaga foetida* |
| CgdH | AMW06315 | *Gemmatimonas phototrophica* |
| CgdH | BAH40890 | *Gemmatimonas aurantiaca* |
| CgdH | AFS53101 | *Leptospirillum ferriphilum* |
| CgdH | CUW40578 | *Magnetospirillum sp.* |
| CgdH | WP_010883524 | *Chlamydia pneumoniae* |
| CgdH | WP_021756895 | *Chlamydia pecorum* |
| CgdH | CAH07682 | *Bacteroides fragilis* |
| CgdH | ACB73891 | *Opitutus terrae* |
| CgdH | ACM93156 | *Nautilia profundicola* |
| CgdH | KIN92132 | *Thauera sp.* |
| CgdH | CUI82205 | *Achromobacter sp.* |
| CgdH | WP_025305675 | *Thermocrinis ruber* |
| CgdH | WP_012674071 | *Sulfurihydrogenibium azorense* |
| CgdH | EHC13895 | *Fischerella sp.* |
| CgdH | WP_036489441 | *Myxosarcina sp.* |
| CgdH | AFZ05950 | *Oscillatoria nigro-viridis* |
| CgdH | P74132 | *Synechocystis* |
| ChuW | AIU70349 | *Thermococcus eurythermalis* |
| ChuW | WP_051366200 | *Pseudothermotoga elfii* |
| ChuW | CDC62198 | *Clostridium sp.* |
| ChuW | WP_020612921 | *Sediminispirochaeta bajacaliforniensis* |
| ChuW | EEF14474 | *Campylobacter rectus* |
| ChuW | WP_013010134 | *Denitrovibrio acetiphilus* |
| ChuW | WP_009353669 | *Veillonella sp.* |
| ChuW | WP_051212161 | *Veillonella montpellierensis* |
| ChuW | EKX99441 | *Selenomonas sp.* |
| ChuW | WP_019177805 | *Methanomassiliicoccus luminyensis* |
| ChuW | WP_013506625 | *Desulfurispirillum indicum* |
| ChuW | EGW43871 | *Bilophila sp.* |
| ChuW | EIO71851 | *Escherichia coli* |
| ChuW | WP_004344082 | *Thauera linaloolentis* |
| ChuW | WP_008619339 | *Magnetospirillum caucaseum* |
| HutW | AIC83437 | *Vibrio alginolyticus* |
| Zbamorf26 | ACG60749 | *Streptomyces pilosus* |
| Blmorf8 | ABL74954 | *Streptoalloteichus hindustanus* |
| (continued) | | |
| Type of CgdH | **Accession_No.** | **Strain** |
| Tlmorf1 | AAG02372 | *Streptomyces verticillus* |
| Class_C_RSM | EGG43722 | *Streptomyces griseoaurantiacus* |
| NosN | ADR01089 | *Nocardia* |
| PbtM3 | AGY49586 | *Planobispora rosea* |
| TpdU | ACS83765 | *Nonomuraea* |
| TbtI | YP_003651165 | *Thermobispora bispora* |
| TpdL | ACS83777 | *Nonomuraea sp.* |
| Class_C_RSM | AGY49595 | *Planobispora rosea* |
| Tpdx2 | SEL93961 | *Nonomuraea pusilla* |
| Class_C_RSM | SDP98233 | *Lentzea jiangxiensis* |
| Class_C_RSM | OGO33555 | *Chloroflexi bacterium* |
| Class_C_RSM | WP_067157183 | *Streptomyces sp.* |
| Jaw5 | BAO98806 | *Streptomyces roseoverticillatus* |
| Class_C_RSM | SEF54573 | *Streptomyces yanglinensis* |
| Class_C_RSM | WP_011291186 | *Thermobifida fusca* |
| Class_C_RSM | GAD86125 | *Nocardia asteroides* |
| Class_C_RSM | WP_026404953 | *Actinomadura rifamycini* |
| Class_C_RSM | WP_030684647 | *Streptomyces sp.* |
| C10P | WP_012324632 | *Shewanella* |
| Class_C_RSM | WP_051685090 | *Clostridium sp.* |
| YtkT | WP_055490826 | *Streptomyces sp.* |
| Class_C_RSM | WP_014677041 | *Streptomyces* |
| HemZ | WP_025117272 | *Lysinibacillus fusiformis* |
| HemZ | Q796V8 | *Bacillus subtilis* |
| HemZ | CON04352 | *Streptococcus pneumoniae* |
| HemZ | WP_010233508 | *Clostridium arbusti* |
| HemZ | WP_007286255 | *Intestinibacter bartlettii* |
| HemZ | WP_039679463 | *Terrisporobacter othiniensis* |
| MenK | AFK50646 | *Thermogladius calderae* |
| MenK | ESQ25637 | *Acidilobus sp.* |
| MenK | AFZ70518 | *Caldisphaera lagunensis* |
| MenK | CCC80926 | *Thermoproteus tenax* |
| MenK | ABM80220 | *Hyperthermus butylicus* |
| MenK | WP_055408506 | *Pyrodictium delaneyi* |
| MenK | AEM37888 | *Pyrolobus fumarii* |
| MenK | WP_011998375 | *Ignicoccus hospitalis* |
| MenK | WP_014025565 | *Pyrolobus fumarii* |
| MenK | WP_011138079 | *Wolinella succinogenes* |
| MenK | WP_028766718 | *Shewanella fidelis* |
| MenK | EGG57223 | *Parasutterella excrementihominis* |
| MenK | CBL03906 | *Gordonibacter pamelaeae* |
| (continued) | | |
| Type of CgdH | **Accession_No.** | **Strain** |
| Plant | XP_011398951 | *Auxenochlorella protothecoides* |
| Plant | XP_005649356 | *Coccomyxa subellipsoidea* |
| Plant | XP_001703480 | *Chlamydomonas reinhardtii* |
| Plant | XP_002946784 | *Volvox carteri* |
| Plant | CAL50029 | *Ostreococcus tauri* |
| Plant | XP_002507726 | *Micromonas commoda* |
| Plant | EDQ75811 | *Physcomitrium patens* |
| Plant | EFJ27703 | *Selaginella moellendorffii* |
| Plant | XP_011627754 | *Amborella trichopoda* |
| Plant | ABI93924 | *Arabidopsis thaliana* |
| Plant | XP_002262672 | *Vitis vinifera* |
| Plant | XP_012487614 | *Gossypium raimondii* |
| Plant | XP_003522422 | *Glycine max* |
| Plant | XP_009616975 | *Nicotiana tomentosiformis* |
| Plant | XP_010250874 | *Nelumbo nucifera* |
| Plant | BAF29564 | *Oryza sativa* |
| Plant | XP_009415069 | *Musa acuminata* |
| Plant | XP_010930027 | *Elaeis guineensis* |
| RSAD1 | EEC11362 | *Ixodes scapularis* |
| RSAD1 | ESA03321 | *Rhizophagus irregularis* |
| RSAD1 | CEP17932 | *Parasitella parasitica* |
| RSAD1 | CEG73035 | *Rhizopus microsporus* |
| RSAD1 | CDS13916 | *Lichtheimia ramosa* |
| RSAD1 | EDV19558 | *Trichoplax adhaerens* |
| RSAD1 | XP_012557630 | *Hydra vulgaris* |
| RSAD1 | HXP_001636936 | *Nematostella vectensis* |
| RSAD1 | EFX89896 | *Daphnia pulex* |
| RSAD1 | ELT99882 | *Capitella teleta* |
| RSAD1 | XP_009064549 | *Lottia gigantea* |
| RSAD1 | XP_005090240 | *Aplysia californica* |
| RSAD1 | XP_012695163 | *Clupea harengus* |
| RSAD1 | NP_001077026 | *Danio rerio* |
| RSAD1 | XP_010862851 | *Esox lucius* |
| RSAD1 | XP_007556327 | *Poecilia formosa* |
| RSAD1 | XP_008327612 | *Cynoglossus semilaevis* |
| RSAD1 | XP_005999660 | *Latimeria chalumnae* |
| RSAD1 | OCA21266 | *Xenopus tropicalis* |
| RSAD1 | XP_007441654 | *Python bivittatus* |
| RSAD1 | XP_008102717 | *Anolis carolinensis* |
| RSAD1 | XP_005297689 | *Chrysemys picta* |
| RSAD1 | XP_009562321 | *Cuculus canorus* |
| RSAD1 | KFV63667 | *Dryobates pubescens* |
| (continued) | | |
| Type of CgdH | **Accession_No.** | **Strain** |
| RSAD1 | XP_005144383 | *Melopsittacus undulatus* |
| RSAD1 | XP_007505827 | *Monodelphis domestica* |
| RSAD1 | XP_012405492 | *Sarcophilus harrisii* |
| RSAD1 | XP_012581510 | *Condylura cristata* |
| RSAD1 | NP_001013399 | *Mus musculus* |
| RSAD1 | XP_008516193 | *Equus przewalskii* |
| RSAD1 | XP_011228768 | *Ailuropoda melanoleuca* |
| RSAD1 | XP_004446612 | *Dasypus novemcinctus* |
| RSAD1 | XP_012518368 | *Propithecus coquereli* |
| RSAD1 | EAW94610 | *Homo sapiens* |
| RSAD1 | XP_003414682 | *Loxodonta africana* |
| RSAD1 | XP_007940360 | *Orycteropus afer* |
| HemW | ACD05922 | *Akkermansia muciniphila* |
| HemW | ALA58796 | *Nitrospira moscoviensis* |
| HemW | CUW28423 | *Streptomyces reticuli* |
| HemW | KOX89034 | *Thermus aquaticus* |
| HemW | WP_052195205 | *Deinococcus radiopugnans* |
| HemW | ADI13526 | *Truepera radiovictrix* |
| HemW | WP_062189862 | *Anaerolinea thermolimosa* |
| HemW | EFH90847 | *Ktedonobacter racemifer* |
| HemW | WP_014433607 | *Caldilinea aerophila* |
| HemW | ACM05800 | *Thermomicrobium roseum* |
| HemW | WP_039744988 | *Geobacter pickeringii* |
| HemW | ACU91423 | *Desulfomicrobium baculatum* |
| HemW | EFV43164 | *Bilophila wadsworthia* |
| HemW | WP_015946007 | *Desulfovibrio vulgaris* |
| HemW | WP_015898519 | *Acidobacterium capsulatum* |
| HemW | ACX75702 | *Fibrobacter succinogenes* |
| HemW | EDM26343 | *Lentisphaera araneosa* |
| HemW | WP_000239943 | *Enterobacteriaceae* |
| Other proteins | WP_013008727 | *Deferribacter desulfuricans* |
| Other proteins | ALD66217 | *Spiroplasma cantharicola* |
| Other proteins | AFG37251 | *Spirochaeta africana* |
| Other proteins | ACV39949 | *Leptotrichia buccalis* |
| Other proteins | ADO82048 | *Ilyobacter polytropus* |
| Other proteins | ERT66913 | *Cetobacterium somerae* |
| Other proteins | ACK42388 | *Dictyoglomus turgidum* |
| Other proteins | ACI19107 | *Dictyoglomus thermophilum* |
| Other proteins | EGG81213 | *Lachnospiraceae bacterium* |
| Other proteins | AGB18790 | *Thermoanaerobacterium thermosaccharolyticum* |
| Other proteins | WP_036947005 | *Pseudobacteroides cellulosolvens* |
| Other proteins | YP_001255455 | *Clostridium botulinum* |
| (continued) | | |
| Type of CgdH | **Accession_No.** | **Strain** |
| Other proteins | CAB61616 | *Bacillus subtilis* |
| Other proteins | WP_010735215 | *Enterococcus mundtii* |
| Other proteins | WP_038057790 | *Thermodesulfobacterium hydrogeniphilum* |
| Other proteins | AIH03372 | *Thermodesulfobacterium commune* |
| Other proteins | WP_009962340 | *Verrucomicrobium spinosum* |
| Other proteins | EAQ77478 | *Blastopirellula marina* |
| Other proteins | KLU03830 | *Rhodopirellula islandica* |
| Other proteins | ADB19145 | *Pirellula staleyi* |
| CgdH1 | YP_002344387 | *Campylobacter jejuni* |
| CgdH2 | YP_002343800 | *Campylobacter jejuni* |
| CgdH3 | YP_002344010 | *Campylobacter jejuni* |
